# Fatty acid ethyl ester synthase inhibition ameliorates ethanol-induced Ca^2+^-dependent mitochondrial dysfunction and acute pancreatitis

**DOI:** 10.1136/gutjnl-2012-304058

**Published:** 2013-10-25

**Authors:** Wei Huang, David M Booth, Matthew C Cane, Michael Chvanov, Muhammad A Javed, Victoria L Elliott, Jane A Armstrong, Hayley Dingsdale, Nicole Cash, Yan Li, William Greenhalf, Rajarshi Mukherjee, Bhupendra S Kaphalia, Mohammed Jaffar, Ole H Petersen, Alexei V Tepikin, Robert Sutton, David N Criddle

**Affiliations:** 1 Department of Cellular & Molecular Physiology, Institute of Translational Medicine, University of Liverpool, Liverpool, Merseyside, UK; 2 NIHR Liverpool Pancreas Biomedical Research Unit, RLBUHT, Institute of Translational Medicine, University of Liverpool, Liverpool, Merseyside, UK; 3 Department of Integrated Traditional Chinese and Western Medicine, Sichuan Provincial Pancreatitis Centre, West China Hospital, Sichuan University, China; 4 Department of Pathology, University of Texas Medical Branch, Galveston, Texas, USA; 5 Morvus Technology Limited, Carmarthen, UK; 6 Cardiff School of Biosciences, University of Cardiff, Cardiff, UK

**Keywords:** ACUTE Pancreatitis, Alcohol-Induced Injury, Calcium, Ethanol, Pancreatic Damage

## Abstract

**Objective:**

Non-oxidative metabolism of ethanol (NOME) produces fatty acid ethyl esters (FAEEs) via carboxylester lipase (CEL) and other enzyme action implicated in mitochondrial injury and acute pancreatitis (AP). This study investigated the relative importance of oxidative and non-oxidative pathways in mitochondrial dysfunction, pancreatic damage and development of alcoholic AP, and whether deleterious effects of NOME are preventable.

**Design:**

Intracellular calcium ([Ca^2+^]_C_), NAD(P)H, mitochondrial membrane potential and activation of apoptotic and necrotic cell death pathways were examined in isolated pancreatic acinar cells in response to ethanol and/or palmitoleic acid (POA) in the presence or absence of 4-methylpyrazole (4-MP) to inhibit oxidative metabolism. A novel in vivo model of alcoholic AP induced by intraperitoneal administration of ethanol and POA was developed to assess the effects of manipulating alcohol metabolism.

**Results:**

Inhibition of OME with 4-MP converted predominantly transient [Ca^2+^]_C_ rises induced by low ethanol/POA combination to sustained elevations, with concurrent mitochondrial depolarisation, fall of NAD(P)H and cellular necrosis in vitro. All effects were prevented by 3-benzyl-6-chloro-2-pyrone (3-BCP), a CEL inhibitor. 3-BCP also significantly inhibited rises of pancreatic FAEE in vivo and ameliorated acute pancreatic damage and inflammation induced by administration of ethanol and POA to mice.

**Conclusions:**

A combination of low ethanol and fatty acid that did not exert deleterious effects per se became toxic when oxidative metabolism was inhibited. The in vitro and in vivo damage was markedly inhibited by blockade of CEL, indicating the potential for development of specific therapy for treatment of alcoholic AP via inhibition of FAEE generation.

**Significance of this study**

**What is already known about this subject?**

- Acute alcoholic pancreatitis can be a severe and life-threatening disease, which currently lacks a clear target and specific therapy.
- Both oxidative and non-oxidative ethanol metabolites have been implicated in ethanol-mediated damage of the exocrine pancreas, although their relative contributions to pancreatic toxicity remain unclear.
- Increasing evidence has implicated mitochondrial dysfunction as a key event in the pathophysiology of acute pancreatitis.
- Currently there is no convenient and reliable in vivo experimental model of alcoholic acute pancreatitis to test potential therapies.

**What are the new findings?**

- A new model of alcoholic acute pancreatitis has been developed.
- Promotion of non-oxidative ethanol metabolism via suppression of the oxidative pathway exacerbated ethanol-induced Ca^2+^-dependent mitochondrial dysfunction and toxicity in vitro and acute pancreatitis in vivo.
- Non-oxidative ethanol metabolites localised to mitochondria of pancreatic acinar cells and underwent hydrolysis to release free fatty acids.
- Pharmacological inhibition of carboxylester lipase (CEL) prevented fatty acid ethyl ester formation and ameliorated in vitro exocrine pancreatic damage and in vivo acute pancreatitis induced by fat and ethanol.

**How might it impact on clinical practice in the foreseeable future?**

- New insights into the underlying pathology of acute alcoholic pancreatitis reveal a specific enzyme target (CEL) that may be inhibited to ameliorate ethanol-induced injury.
- Elucidation of the molecular basis of non-oxidative ethanol metabolism mediated by CEL and inhibition by 3-benzyl-6-chloro-2-pyrone (3-BCP) will facilitate rational design of novel therapeutic compounds that may now be evaluated using a convenient and reliable experimental model of acute alcoholic pancreatitis.

## Introduction

In recent decades there has been a marked elevation in alcohol consumption mirrored by a dramatic increase in the incidence of acute pancreatitis (AP). Approximately 20% of affected individuals develop extensive disease characterised by significant pancreatic necrosis and systemic inflammation, which may lead to multiple organ failure and death. Our understanding of the pathogenesis of alcoholic AP remains incomplete and currently no specific therapy exists.

The exocrine pancreas uses both oxidative and non-oxidative metabolism of ethanol (OME and NOME, respectively) to degrade alcohol, the latter combining ethanol with fatty acids to yield lipophilic fatty acid ethyl esters (FAEEs).^1^ A seminal study showed that FAEEs accumulated in the pancreas of patients following acute alcohol intoxication, in contrast to other organs commonly damaged by alcohol.^2^ The balance between OME and NOME may be pivotal in determining toxic effects of excess alcohol. For example, blockade of OME by 4-methylpyrazole (4-MP) increased pancreatic FAEE levels of rats in response to ethanol, resulting in organ damage and inflammation.^3^

The generation of FAEEs occurs via diverse FAEE synthase enzymes^4^ that include carboxylester lipase (CEL; E.C.3.1.1.13), also known as bile salt-activated lipase,^5^ inhibited by 3-benzyl-6-chloro-2-pyrone (3-BCP).^6^ CEL is synthesised by pancreatic acinar cells (PACs), stored in zymogen granules, and secreted upon hormonal stimulation.^7^ Long-term ethanol feeding increased pancreatic CEL in rats,^8^ while high FAEE concentrations remain in the serum of patients after ethanol ingestion,^9^ persisting for up to 99 h in heavy drinkers.^10^ Raised levels (>5× control) occur in the serum of patients with acute interstitial or necrotising pancreatitis.^11^

We previously demonstrated that FAEEs induced toxic sustained [Ca^2+^]_C_ elevations in PACs that led to necrosis.^12^ Mitochondrial dysfunction was central to damage; Ca^2+^ overload caused loss of membrane potential (Δψ_M_) and consequent ATP fall, which impaired [Ca^2+^]_C_ homeostasis. Supply of ATP to the cell via patch-pipette inhibited development of toxic [Ca^2+^]_C_ elevations and necrosis induced by FAEEs^13^ and bile acids.^14^ Nevertheless, supply of ATP to stressed cells is unlikely to be a viable strategy for prevention of alcohol-induced toxicity in a clinical setting and alternative approaches are necessary.

This study has investigated whether manipulation of alcohol metabolic pathways may influence toxicity in vitro and the development of AP in vivo, and if inhibition of CEL with 3-BCP could prevent deleterious effects. Using molecular modelling, we demonstrate the likely mechanism of CEL-mediated FAEE generation and mode of 3-BCP inhibition. Our findings show that NOME promotion, in response to low ethanol/palmitoleic acid (POA) with concurrent OME inhibition, induced Ca^2+^-dependent mitochondrial dysfunction and PAC necrosis. Furthermore, we have developed a novel experimental model of alcoholic AP based on the combination of ethanol and POA to induce acute exocrine pancreatic damage and systemic inflammation. Both in vitro and in vivo deleterious effects of ethanol/POA associated with promotion of NOME were ameliorated by CEL inhibition, suggesting potential for therapeutic intervention in alcoholic AP.

## Materials and methods

### Cell preparation and solutions

Isolated PACs from adult CD1 mice were prepared as previously described.^13^ Experiments were performed at room temperature unless otherwise indicated. Extracellular solution contained (mM): 140 NaCl, 4.7 KCl, 1.13 MgCl_2_, 1 CaCl_2_, 10 D-glucose, 10 HEPES (adjusted to pH 7.35 using NaOH).

### Measurements of [Ca^2+^]_C_, Δψ_M_ and NAD(P)H

Confocal imaging was performed using a Zeiss LSM510 system (Jena GmbH, Germany) at 35°C. Cells were loaded with Fluo 4-AM (3 μM; excitation 488 nm, emission 505–550 nm) or tetramethyl rhodamine methyl ester (TMRM, 100 nM; excitation 488 nm, emission >550 nm) to measure [Ca^2+^]_C_ and Δψ_M_, respectively, and mitochondrial metabolism (NAD(P)H autofluorescence; excitation 363 nm, emission 390–450 nm) assessed as described.^13^ Image analysis was performed using AIM V.4.2 or Zen2009 software. Ethanol (10 mM), POA (20 µM), 4-MP (100 µM) and 3-BCP (10 µM) were applied alone or in combination for 10 min, after which the protonophore carbonyl cyanide 3-chlorophenylhydrazone (CCCP, 10 µM) was added to induce complete mitochondrial depolarisation. Measurements are expressed as changes from basal fluorescence (F/F_0_ ratio, ‘n’ represents number of cells).

### Detection of apoptotic and necrotic cell death pathway activation

Apoptotic cell death pathway activation was detected using rhodamine 110-aspartic acid amide (20 μM: excitation 488 nm, emission 505–550 nm), necrotic cell death pathway activation using propidium iodide (PI, 1 µM: excitation 488 nm, emission 630–693 nm) and total cell count with nuclear Hoechst 33342 (10 µg/mL: excitation 364 nm, emission 405–450 nm) as described.^14^ Separate cell aliquots from each preparation were treated (30 min) with either (i) ethanol (10 mM), (ii) POA (20 µM), (iii) 4-MP (100 µM) or (iv) 3-BCP (10 µM) or combinations to differentially modulate OME and NOME. Counts were made of 30 separate fields from each experiment, repeated in triplicate or more.

### Immunofluorescence

Intact pancreatic lobules or isolated acinar cells were fixed in 4% paraformaldehyde for 30 min, permeabilised with 0.2% Triton X-100 and blocked with 1% BSA/10% goat serum. CEL and mitochondria were localised using anti-bile salt-activated lipase antibody and mitochondrial anti-peptidyl-prolyl cis-trans isomerase antibody, respectively (both 1:200, Abcam, Cambridge UK). Secondary antibodies were conjugated with Alexa 488 or Alexa 546, used at a dilution of 1:1000. Nuclei were stained with Hoechst 33342 (10 µg/mL, Invitrogen). CEL antibody specificity was confirmed by Western blot (see online supplementary figure S2B).

### Patch-clamp current recording

Patch-clamp (whole-cell configuration) was used to record Ca^2+^-activated Cl^−^ currents (ICl_Ca_) as described.^13^ Patch-pipettes (2–3 MΩ) were filled with intracellular solution (mM): 140 KCl; 1.5 MgCl_2_; 2 MgATP; 10 HEPES; 0.1 EGTA, pH 7.2. Currents were sampled at 10 KHz (EPC8 amplifier, HEKA, Lambrecht, Pfalz, Germany) with membrane voltage clamped at −30 mV. Changes of ICl_Ca_, NAD(P)H and fluorescein (excitation 488 nm, emission 505–550 nm) were recorded, before and after exposure to POA-Fluor (20 µM: in pipette solution).

### In vivo model of FAEE-AP

To establish FAEE-induced AP (FAEE-AP), adult CD1 mice received two intraperitoneal injections of ethanol (1.35 g/kg) and POA (150 mg/kg) at 1 h intervals. 200 µL normal saline was injected immediately prior to ethanol/POA injections to avoid potential local damage by ethanol to peritoneal organs at the injection site. Control adult CD1 mice received either saline, ethanol (1.35 g/kg) or POA (150 mg/kg). In treatment groups, mice also received 10 mg/kg 4-MP or 30 mg/kg 3-BCP simultaneously with the first injection of ethanol/POA. Animals were sacrificed at 24 h after the first injection. Histological assessment of damage was performed after H&E staining of fixed pancreatic slices (5 µm thickness); 10 random fields per slide from all animal groups were graded by two independent, blinded observers according to severity and extent of oedema, inflammatory cell infiltration and acinar necrosis as previously described.^15^ Further details of FAEE-AP model characterisation are included in online supplementary materials and methods.

### Computational protein-ligand docking studies of POA and 3-BCP

Three-dimensional structures of ligands (POA and 3-BCP) were constructed (ChemDraw; V.11.0) and energy minimised. The X-ray crystallographic co-ordinates of human CEL (pdb 1F6W) were downloaded from RCSPB Protein Database and used for docking procedures. Molegro Virtual Docker (MVD V.3) was used to predict ligand binding mode in the protein active site. MolDock optimiser algorithms were used with a maximum of 10 runs and 10 000 iterations per ligand. A maximum of five poses were returned per ligand in which similar poses were clustered.^16^ The top-ranked pose for each ligand was assigned with respect to affinity (hydrogen bond and other residue interactions, which may contribute to stability in the active site). The ligand-protein interactions were visualised using Molegro Molecular Viewer (V.2.2).

### Statistical analysis

Statistical analysis of variance was performed using Origin 8.5. A value of p<0.05 was considered significant.

### Chemicals

Fluo 4-AM, TMRM, secondary antibodies, Hoechst 33342 and R110-aspartic acid amide were purchased from Molecular Probes (Eugene, Oregon, USA). POA-Fluor was synthesised by Dr M Jaffar as described (see online supplementary methods and figure S1) and 3-BCP by Dr B S Kaphalia.^17^ All other chemicals were purchased from Sigma (Gillingham, UK).

## Results

### Inhibition of OME transforms transient [Ca^2+^]_C_ signals induced by ethanol and POA to sustained elevations: protective effects of 3-BCP

**Figure 1:**
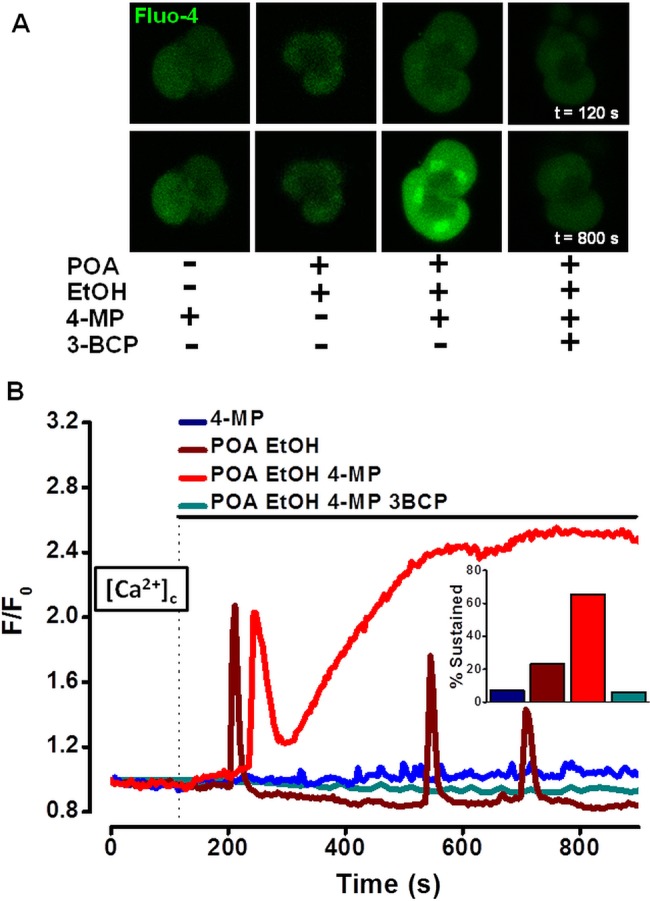
Effects of ethanol (EtOH), palmitoleic acid (POA), 4-methylpyrazole (4-MP) and 3-benzyl-6-chloro-2-pyrone (3-BCP) on [Ca^2+^]_C_ in pancreatic acinar cells. (A) Typical fluorescence images showing changes of [Ca^2+^]_C_ (Fluo4, *green*) before (t=120 s) and after (t=800 s) application of combinations of ethanol (10 mmol/L), POA (20 μmol/L), 4-MP (100 μmol/L) and 3-BCP (10 μmol/L). Addition of ethanol/POA/4-MP caused sustained elevation of [Ca^2+^]_c_ seen at 800 s. (B) Combination of ethanol/POA (*wine*) induced predominantly oscillatory increases of [Ca^2+^]_C_, while additional presence of 4-MP (100 μmol/L) promoted a shift towards sustained increases (*red*); 4-MP alone was without effect (*blue*). The addition of 3-BCP abolished sustained rises induced by ethanol/POA/4-MP (*cyan*). Cumulative data expressed as percentage of cells exhibiting sustained rises (inset) (F/F_0_=>1.5) at 800 s (total n=159 cells).

In PACs, application of ethanol (10 mM) with POA (20 µM) produced predominantly transient [Ca^2+^]_C_ elevations (32 of 52 cells*,* 61.5%), with sustained signals observed in only 23.1% (12 of 52 cells, figure 1A,B). The OME inhibitor 4-MP produced no or minimal effects on [Ca^2+^]_C_ per se, but greatly potentiated ethanol/POA action, increasing the proportion of sustained [Ca^2+^]_C_ elevations to 65.5% (figure 1B inset). Addition of 3-BCP (10 µM), which produced no effects on resting [Ca^2+^]_C_ alone (see online supplementary figure S7A), completely blocked the increase in sustained [Ca^2+^]_C_ elevations induced by ethanol/POA/4-MP (figure 1B). The inhibitory effects of 3-BCP were specific to ethanol/POA mediated changes, since oscillatory or sustained [Ca^2+^]_C_ elevations induced by 10 pM and 10 nM cholecystokinin, respectively, were unaffected (see online supplementary figure S7B,C).

### Inhibition of OME exacerbates loss of Δψ_m_ and fall of NAD(P)H induced by EtOH and POA: protective effects of 3-BCP

**Figure 2:**
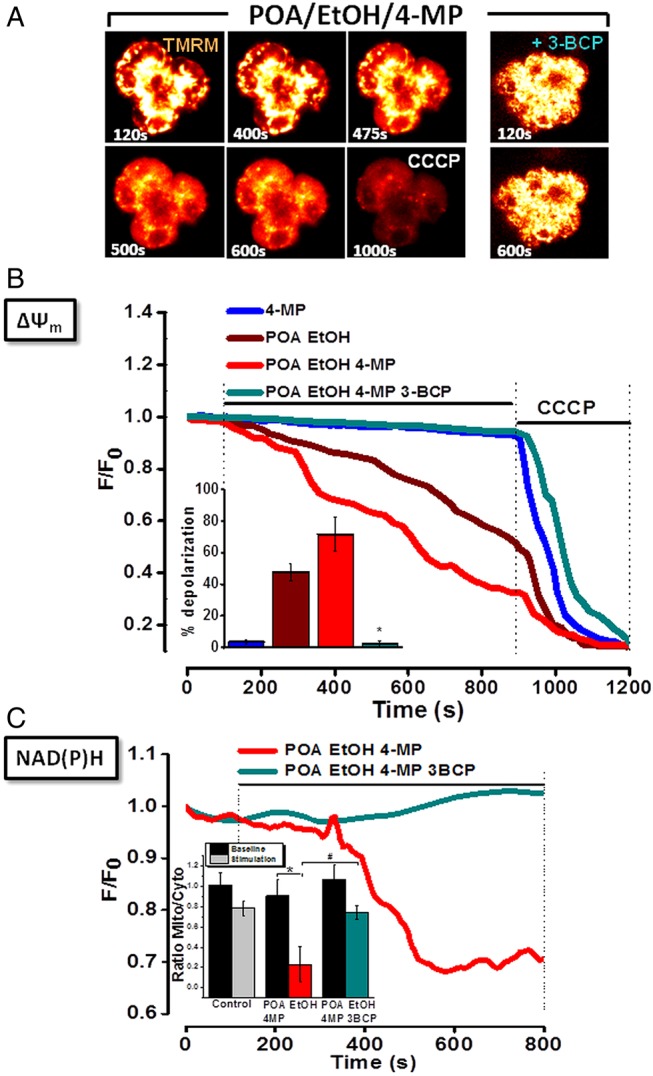
Effects of ethanol (EtOH), palmitoleic acid (POA), 4-methylpyrazole (4-MP) and 3-benzyl-6-chloro-2-pyrone (3-BCP) on mitochondrial membrane potential (Δψ_M_) in pancreatic acinar cells. (*A*) Typical fluorescence (*glow*) images showing progressive loss (six images, *left*) or maintenance (two images, *right*) of Δψ_M_ to ethanol/POA/4-MP combination in absence (*left*), or presence (*right*) of 3-BCP. Complete mitochondrial depolarisation induced by carbonyl cyanide 3-chlorophenylhydrazone (CCCP;10 μmol/L) is seen at 900 s. (B) Graph showing ethanol/POA-induced loss of Δψ_M_ (n=39), exacerbated by 4-MP (n=44). 4-MP alone was without effect (n=23). 3-BCP abolished effects of ethanol/POA/4-MP (n=36). Summarised data (inset) show mean % depolarisation±SE for each application. (C) Progressive loss of mitochondrial reduced nicotinamide adenine dinucleotide (phosphate) (NAD(P)H) fluorescence induced by ethanol/POA/4-MP (*red*, n=44) was abolished by 3-BCP (*cyan*, n=36). Summarised data (inset) show changes of NAD(P)H fluorescence, expressed as mean±SE in mitochondria-specific (*mito*) versus mitochondria-free (cytosolic; *cyto*) regions, at baseline (70–100 s) and during stimulation (770–800 s) (* and ^#^ p<0.05 compared to control).

A combination of ethanol (10 mM) and POA (20 µM) induced a gradual mitochondrial depolarisation in PACs. Addition of the protonophore CCCP (10 µM) after ethanol/POA caused a further loss of fluorescence as mitochondria became fully depolarised (figure 2A,B). In the additional presence of 4-MP, mitochondrial depolarisation induced by low ethanol/POA was potentiated, whereas 4-MP alone did not alter Δψ_M_. A profound reduction of NAD(P)H autofluorescence in the mitochondrial perigranular region was associated with the loss of Δψ_M_, indicative of mitochondrial inhibition (figure 2C),^14^ an effect more rapid in onset when OME was inhibited (data not shown). 3-BCP (10 µM), which produced no effects on Δψ_M_ or NAD(P)H levels alone (see online supplementary figure S8B,D), completely blocked the depolarisation and fall in NAD(P)H induced by low ethanol/POA/4-MP, although further addition of CCCP depolarised the mitochondria fully, indicating that organellar integrity had not been compromised by the ethanol/POA/4-MP combination. A partial decrease in intracellular ATP levels in response to a combination of ethanol (10 mM) and POA (20 µM) was inhibited by 3-BCP, which did not alter basal levels per se (see online supplementary figure 8E,F). Further addition of CCCP caused a maximal depletion, as shown previously.^13^

### Mitochondrial activation of an FAEE probe induces Ca^2+^-activated Cl^−^ currents (ICl_Ca_) and mitochondrial inhibition

**Figure 3:**
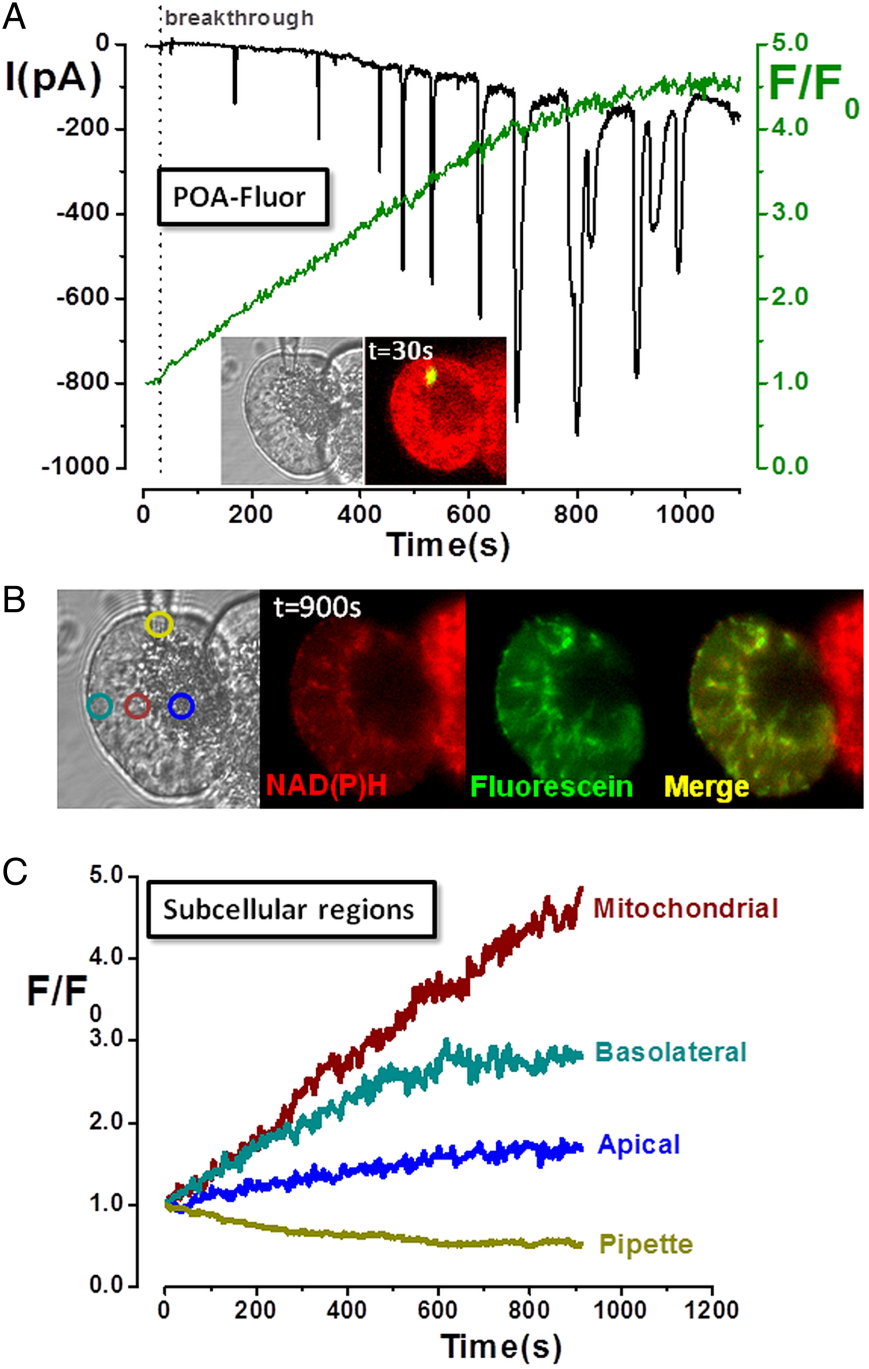
Mitochondrial localisation and activation of fatty acid ethyl ester probe (palmitoleic acid (POA)-Fluor) in pancreatic acinar cells. (A) Light-transmitted and fluorescent images of acinar cell doublet (*inset*) at time of membrane rupture (breakthrough) show localised fluorescence at patch-pipette tip as POA-Fluor activation by hydrolases released fluorescein. Time-dependent fluorescence rise (*green*) was associated with inward Ca^2+^-activated Cl^−^ currents (ICl_Ca_), whereas no fluorescence was detected in the adjacent cell. (B) Light-transmitted and fluorescence images, and (C) subcellular regions of interest show predominantly mitochondrial distribution of fluorescence (fluorescein:NAD(P)H co-localisation), consistent with mitochondrial probe activation. [Ca^2+^]_C_ levels increased over time (sustained inward ICl_Ca_ with superimposed transients), accompanied by a concomitant decrease of NAD(P)H (not seen in non-patched, adjacent cell), consistent with fatty acid-induced mitochondrial inhibition.

Previous biochemical work in cardiomyocytes suggested that FAEEs preferentially accumulate in mitochondria where they are hydrolysed to fatty acids, which uncouple oxidative phosphorylation.^18^ Experiments were performed using a novel probe, POA-Fluor, composed of fluorescein linked to POA via an ester linkage, analogous to that of FAEEs. Although POA-Fluor was non-fluorescent, when administered to the PAC interior via a patch-pipette, cleavage of the probe caused a fluorescence increase as fluorescein and POA were released (figure 3A). Inward ICl_Ca_ developed following probe activation, composed of initial transients that gradually became superimposed on a slowly developing, sustained component, indicating maintained [Ca^2+^]_C_ elevation. The fluorescence rise was greatest in the mitochondrial region (figure 3B,C), suggesting this was the primary site of hydrolysis, with a smaller elevation in the basolateral region, consistent with diffusion of fluorescein. In cells receiving POA-Fluor, NAD(P)H levels decreased as mitochondria were inhibited, in contrast to adjacent cells which maintained their NAD(P)H (figure 3B).

### Protective effects of 3-BCP on pancreatic acinar cell fate

**Figure 4:**
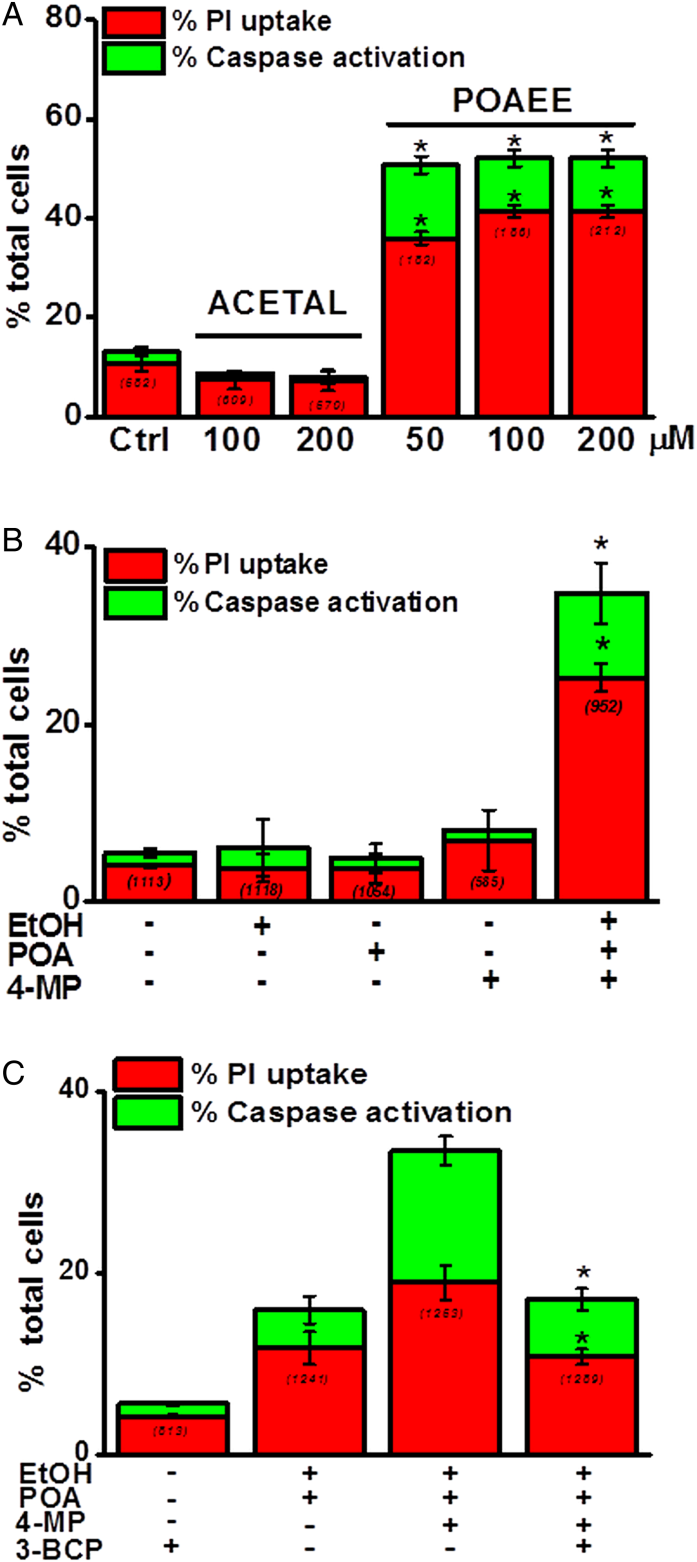
Importance of non-oxidative metabolism of ethanol and oxidative metabolism of ethanol in pancreatic acinar cell death. (A) Levels of apoptosis (caspase; *green*) and necrosis (PI; *red*) were increased by palmitoleic acid ethyl ester (POAEE;50–200 μmol/L) compared with controls, whereas acetaldehyde (ACETAL, 100–200 μmol/L) was without effect. (B) Ethanol (EtOH; 10 mmol/L), palmitoleic acid (POA;20 μmol/L) or 4-methylpyrazole (4-MP;100 μmol/L), applied alone, did not increase caspase activation (*green*) or PI uptake (*red*). However, a combination significantly increased apoptotic and necrotic cell death pathway activation. (C) Inhibition of carboxylester lipase with 3-benzyl-6-chloro-2-pyrone (3-BCP;10 μmol/L) reversed ethanol/POA-induced cell death precipitated by 4-MP (expressed as % total cells; mean±SE; numbers in parentheses indicate cells assessed for each experimental condition, *p<0.05 compared to control values).

Simultaneous detection of apoptotic and necrotic cell death pathways in vitro showed that the NOME metabolite palmitoleic acid ethyl ester (POAEE) (50–200 µM) significantly increased cell death (figure 4A); 200 µM POAEE increased apoptosis from 0.4% (7/582 cells) to 10.8% (23/212 cells) and necrosis from 10.8% (63/582 cells) to 41.5% (88/212 cells). In contrast, application of acetaldehyde (100–200 µM) did not increase either cell death pathway above non-treated controls (figure 4A).

Ethanol (10 mM), POA (20 µM) and 4-MP (100 µM) alone produced no significant increases of either apoptotic or necrotic cell death pathway activation above control values. In contrast, the ethanol/POA/4-MP combination significantly elevated overall cell death: apoptotic cell death rose from 1.4% (16/1113 cells) to 9.5% (90/952 cells) and necrotic cell death from 4.1% (45/1113 cells) to 25.3% (247/952 cells), respectively (figure 4B). In separate experiments, application of ethanol/POA increased cell death, an effect exacerbated by inhibition of OME with 4-MP (100 µM). The CEL inhibitor 3-BCP (10 µM) significantly protected against damage induced by the ethanol/POA/4-MP combination (figure 4C).

### Carboxylester lipase: localisation, formation of POAEE and inhibition by 3-BCP

**Figure 5:**
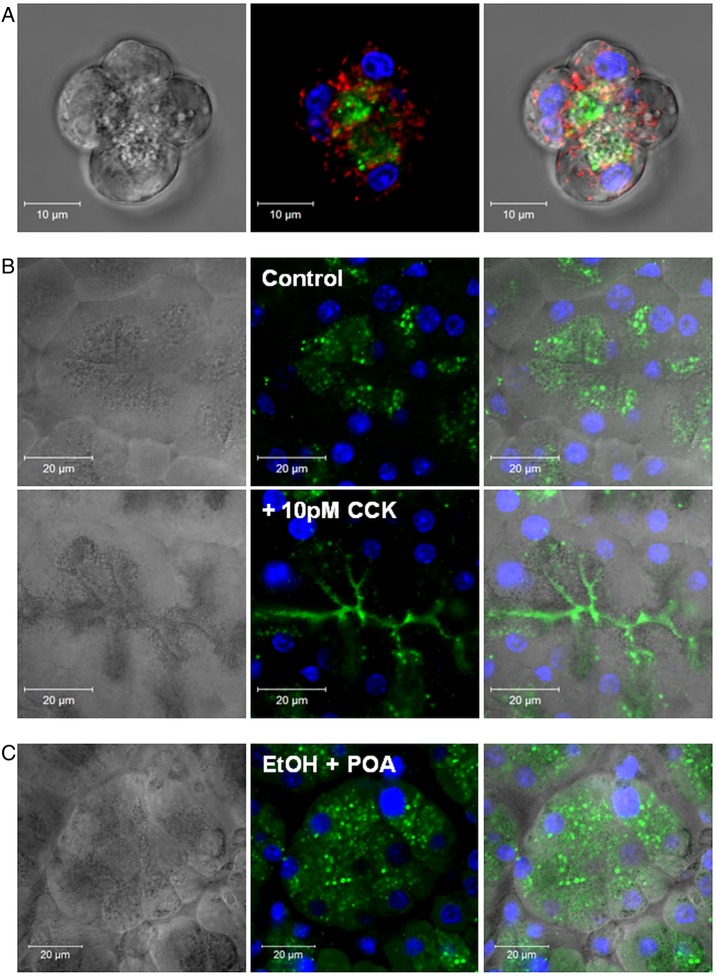
Localisation of carboxylester lipase (CEL) in pancreatic acinar cells and lobules. (A) Light-transmitted and fluorescent images of acinar cells showing CEL (*green*) location in the apical granular region, surrounded by peri-granular mitochondria (mitochondrial peptidyl-prolyl cis-trans isomerase: *red*). Nuclei co-stained with Hoechst 33342 (*blue*). (B) Similar distribution of CEL in unstimulated intact tissue. Treatment with physiological cholecystokinin (10 pM for 30 min) caused CEL to redistribute into the lumen between acinar clusters suggestive of secretion. (C) A diffuse intragranular and extragranular (apical and basolateral) distribution of CEL was observed in intact pancreatic lobules obtained from mice treated with ethanol/POA combination to induce alcoholic acute pancreatitis (i.p. ethanol (1.35 g/kg) and POA (150 mg/kg)).

Immunofluorescence showed predominantly apical, granular distribution of CEL in unstimulated isolated PACs and lobules (figure 5A,B), consistent with prior observations.^19^ However, CEL was redistributed into the luminal space between acinar clusters in response to treatment with physiological cholecystokinin (10 pM), consistent with secretion (figure 5B). Such redistribution into the lumen was also apparent in pancreatic lobules from mice that received caerulein stimulation in vivo (see online supplementary figure S2A). In contrast, widespread intragranular and extragranular CEL distribution, throughout apical and basolateral regions, was observed in pancreatic lobules from mice treated with ethanol/POA to induce in vivo alcoholic AP (figure 5C). The translocation of CEL was observed in areas of tissue damage in the FAEE-AP model as early as 15 min after induction (see online supplementary figure S2C).

**Figure 6:**
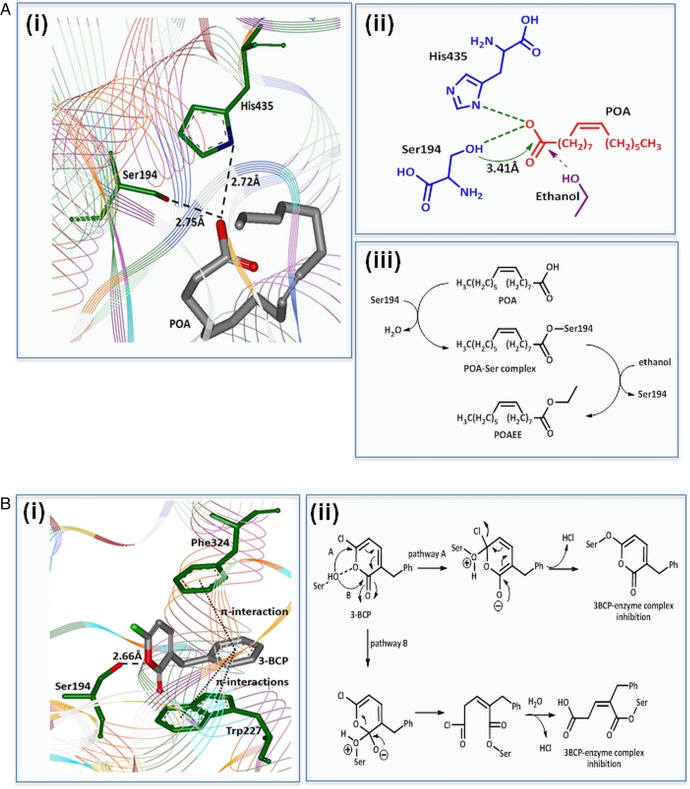
Proposed mechanism of fatty acid ethyl ester (FAEE) generation by carboxylester lipase (CEL) and inhibition by 3-benzyl-6-chloro-2-pyrone (3-BCP). (A) (i) Molecular docking interactions predict binding of palmitoleic acid (POA) in the active site of CEL via interaction with Ser194 and His435 residues suggesting feasibility of mechanism-based esterification. (ii) 2D representation of the binding mode of POA in the active site indicates that the Ser194 hydroxyl group can react with the POA carbonyl group to afford a POA-Ser complex; ethanol easily displaces Ser from the complex forming POA ethyl ester (POAEE). (iii) Mechanism-based esterification of POA to POAEE. (B) (i) Binding of 3-BCP in CEL active site indicates interaction with Ser194 (via hydrogen bonding), further stabilised by two π-interactions with Phe324 and Trp227. (ii) Mechanism-based reaction of 3-BCP with Ser194 residue resulting in formation of 3-BCP-enzyme complex which is inhibitory for FAEE formation.

Computational docking studies indicated strong hydrogen bonding interactions between Ser194 (Ser-OH:HO_2_C-POA; 2.75 Å) and His432 (His-N:HO_2_C-POA, 2.72Å) and the carboxylic acid group of POA in the active site of CEL (figure 6A (i)). Interaction of Ser194 with the carboxylic acid moiety of POA puts it in close enough proximity (<4Å) for covalent reaction (via mechanism-based esterification) resulting in formation of a Ser-POA complex (figure 6A (ii)). The Ser-POA complex can readily react with ethanol to form POAEE (figure 6A (iii)).^20^ Docking results with the CEL inhibitor 3-BCP indicated that it occupies a similar special position to the substrate allowing competition with POA for the active site (figure 6B (i)). 3-BCP is in close proximity to Ser194 (hydrogen bonding between Ser-OH…OCO-3-BCP; 2.66Å) and further stabilised by π-interactions (with Trp227 and Phe324). Moreover, the Ser194 can react covalently with 3-BCP via the C-Cl group (pathway A) or the lactone carbonyl (pathway B) to form 3-BCP-enzyme complexes in a mechanism-based inhibition/inactivation process (figure 6B (ii)).^21^ Confirmation of the inhibitory action of 3-BCP on CEL was shown via a concentration-dependent reduction of the rate of *p*-nitrophenylacetate hydrolysis in isolated PACs (see online supplementary figure S5C).

### Protective effects of 3-BCP in a novel in vivo model of alcoholic AP: FAEE-AP

**Figure 7:**
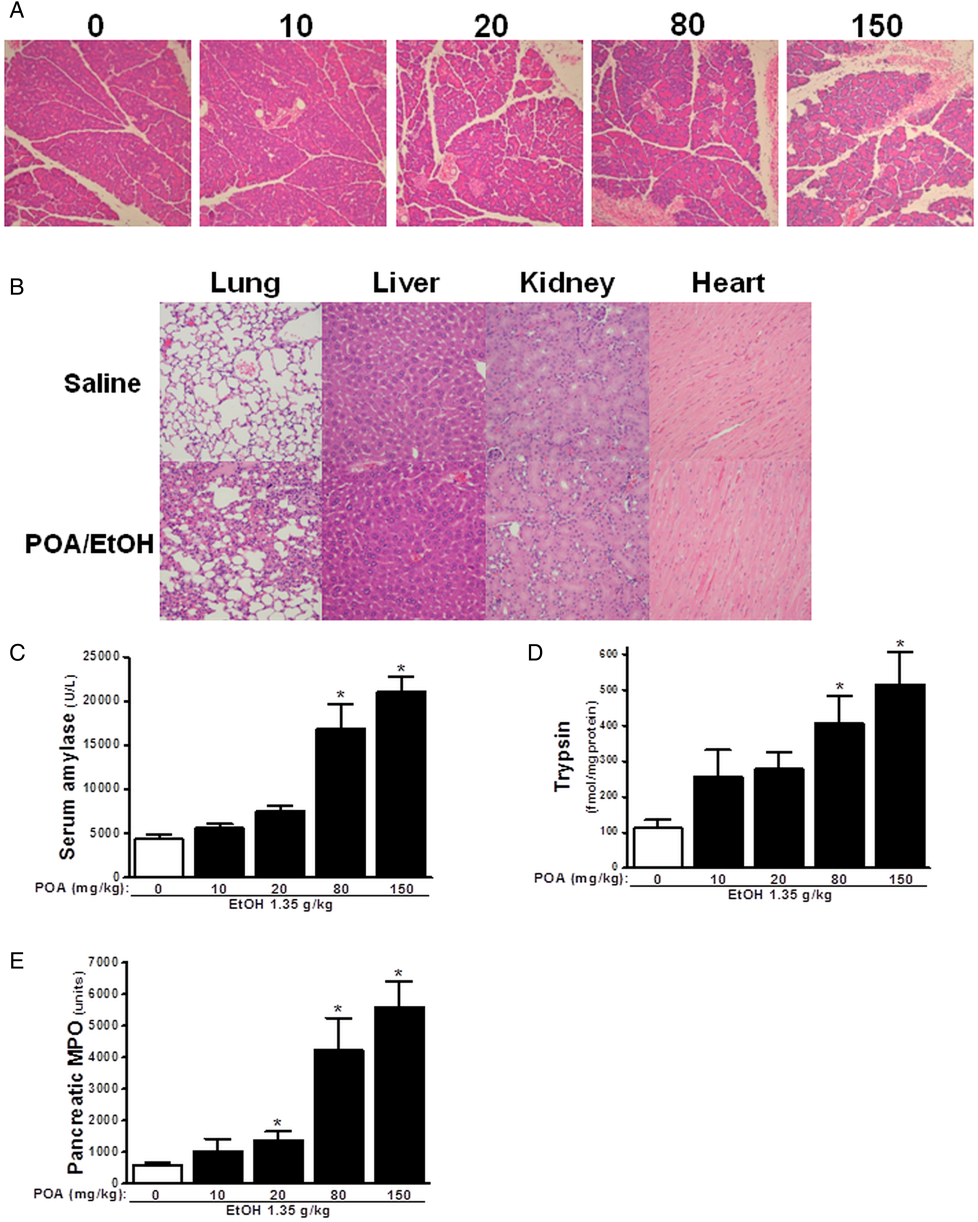
Features of fatty acid ethyl ester-induced acute pancreatitis induced by palmitoleic acid (POA)/ethanol. Mice received two intraperitoneal injections of ethanol (1.35 g/kg) in combination with POA at 0, 10, 20, 80 and 150 mg/kg, and mice were sacrificed 24 h after first injection. (A) Representative H&E images of histology slides from pancreas of mice treated with ethanol with POA. (B) Ethanol with POA (150 mg/kg) caused alveolar membrane thickening and inflammatory infiltration of the lung but did not induce any significant damage to liver, kidney or heart. Effects of ethanol with POA on (C) serum amylase, (D) Pancreatic trypsin activity and (E) Pancreatic myeloperoxidase activity (normalised). *p<0.05 compared to POA alone group. Values are mean±SE of 4–6 mice. Magnification: ×200.

The potential protective effects of 3-BCP were investigated in a novel in vivo model of alcoholic AP. Intraperitoneal injections of ethanol (1.35 g/kg), in combination with POA (0, 10, 20, 80 and 150 mg/kg), induced pancreatic damage observed in histological slices taken 24 h after application, with extensive acinar cell oedema, neutrophil infiltration and necrosis apparent at 80 and 150 mg/kg (figure 7A). The combination of ethanol/POA also elicited alveolar membrane thickening and inflammatory infiltration of the lung but did not damage liver, kidney or heart (figure 7B). Similarly, biochemical markers of AP, including serum amylase, pancreatic trypsin and myeloperoxidase (MPO) activity, were elevated in a dose-dependent manner by the ethanol/POA combination (figure 7C (i–iii)). The time courses of biochemical changes in the FAEE-AP model induced by ethanol and POA are shown in online supplementary materials and figure S3. In a similar manner, AP was induced by direct intraperitoneal application of POAEE, thereby bypassing the endogenous synthetic route or alternatively using oleic acid instead of POA (see online supplementary figure S4).

**Figure 8:**
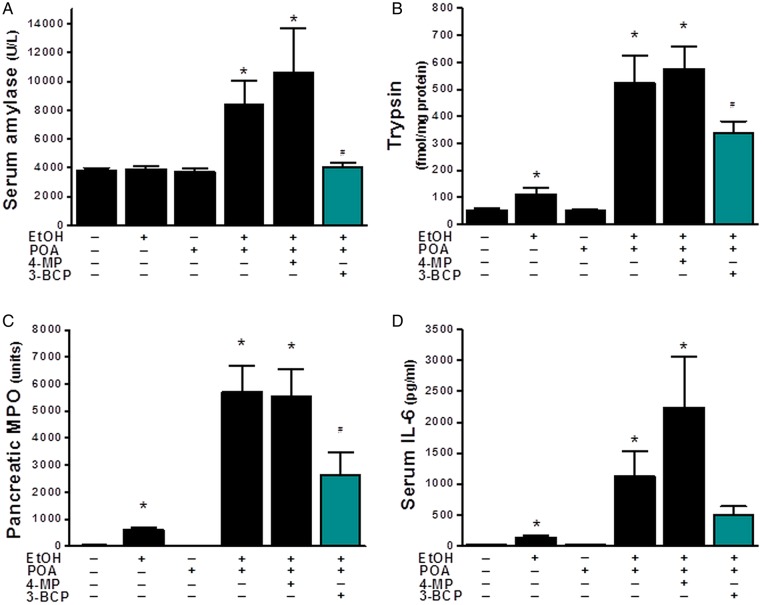
Protective effects of 3-benzyl-6-chloro-2-pyrone (3-BCP) on biomarkers of fatty acid ethyl ester-induced acute pancreatitis (FAEE-AP) induced by palmitoleic acid (POA)/ethanol. FAEE-AP was induced by two intraperitoneal injections of ethanol (1.35 g/kg) and POA (150 mg/kg). The effects of inhibition of oxidative metabolism of ethanol (OME) with 4-methylpyrazole (4-MP; 10 mg/kg) or non-OME with 3-BCP (30 mg/kg), given on the first injection of POA/ethanol, were assessed on (A) Serum amylase, (B) Pancreatic trypsin activity, (C) Pancreatic myeloperoxidase activity (normalised) and (D) Serum interleukin-6 (IL-6). Control mice received saline injections only. Mice were sacrificed 24 h after the first injection. *p<0.05 compared to both saline and POA controls, ^#^p<0.05 compared to the POA/ethanol group. Values are mean±SE of 6 mice.

**Figure 9:**
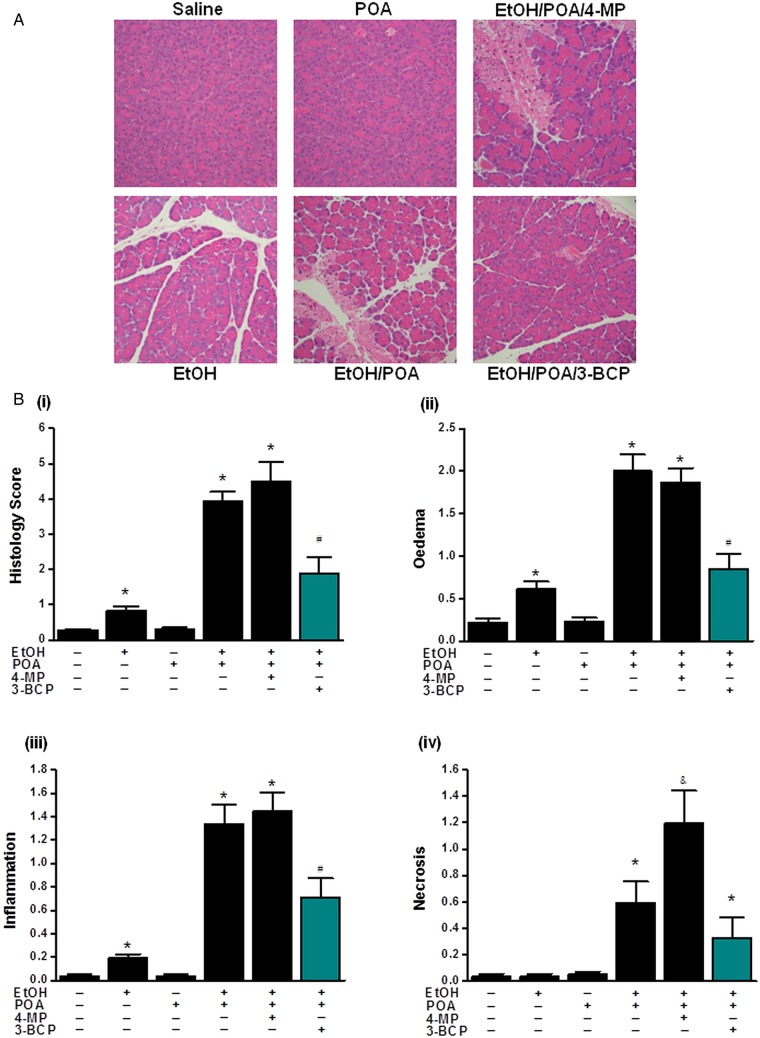
Protective effects 3-benzyl-6-chloro-2-pyrone (3-BCP) on histological parameters of alcoholic acute pancreatitis. (A) Representative H&E images of pancreas histology slides from treatment groups; combinations of ethanol (1.35 g/kg), palmitoleic acid (POA; 150 mg/kg), 4-methylpyrazole (4-MP; 10 mg/kg) and 3-BCP (30 mg/kg). (B)(i) Overall histopathological score and breakdown components: (ii) oedema, (iii) inflammation and (iv) necrosis. All detrimental changes induced by ethanol/POA/4-MP were significantly ameliorated by 3-BCP. (*p<0.05 compared to both saline and POA controls, ^#^p<0.05 compared to ethanol/POA group. Values are mean±SE of 6 mice. Magnification: ×200).

Application of 3-BCP (30 mg/kg) significantly reduced elevations of serum amylase, pancreatic trypsin activity and pancreatic MPO induced by ethanol/POA (figure 8A–D). Similarly, it greatly ameliorated histopathological pancreatic damage induced by ethanol/POA; overall histology score, acinar cell oedema, neutrophil infiltration and necrosis were all significantly reduced by 3-BCP (figure 9A,B (i–iv)). In contrast, ethanol or POA alone caused either a slight or no increase, respectively, in the overall histopathology score (figure 9B (i)). The mild effect of ethanol alone was due to small but significant elevations of neutrophil infiltration and oedema rather than acinar cell necrosis that remained unaltered (figure 9B (ii–iv)). Concurrent inhibition of OME by 4-MP, which by itself did not induce pancreatic damage, caused significantly greater PAC necrosis than that induced by ethanol/POA alone (figure 9B (iv)); however, other parameters of AP were not significantly different (figures 8A–D and 9B (ii–iii)).

An increase in POAEE was detected in the pancreas 30 min after mice received ethanol/POA, which subsequently declined during the following hours, whereas plasma POAEE remained at control levels (see online supplementary figure S5A,B). The elevation of FAEE levels in the pancreas was significantly inhibited by 3-BCP treatment (see online supplementary figure S5A). The protective effects of 3-BCP were specific to FAEE-AP since the CEL inhibitor did not ameliorate AP induced by caerulein hyperstimulation (see online supplementary figure S6A–G).

## Discussion

This study has demonstrated the importance of NOME in mediating acute pancreatic toxicity and inflammation induced by alcohol, damage arising primarily from Ca^2+^-dependent mitochondrial dysfunction in PACs. Many theories have been proposed to explain the detrimental effects of alcohol in AP,^22^ ^23^ including direct actions on Ca^2+^-release mechanisms,^24^ OME-sensitised mitochondrial dysfunction^25^ and formation of toxic FAEEs.^26^ The association between alcohol and fat in pancreatic damage is particularly intriguing; diets rich in corn oil and alcohol induce chronic pancreatic injury in rats.^24^ Epidemiological studies suggest that high fat diets may be linked to development of acute and chronic alcoholic pancreatitis,^27^ while hypertriglyceridaemia is an independent risk factor for both.^28^ The present data highlight the importance of NOME in mediating acute PAC damage leading to AP. It is likely that under conditions of OME inhibition, available alcohol and fatty acid from triglyceride hydrolysis are shunted towards FAEE synthesis via NOME. In anaesthetised rats in which OME was inhibited, the magnitude of FAEE production closely correlated the concentration of ethanol injected intravenously,^3^ while plasma FAEE levels were raised in human subjects following 4-MP treatment.^29^ Diverse FAEEs, including the unsaturated ester POAEE, are detectable in the serum of patients following ethanol ingestion.^9^

Application of exogenous POAEE greatly increased PAC death, while low level ethanol/POA, which did not cause damage per se, became toxic when OME was inhibited. Since this effect was sensitive to 3-BCP, the most likely explanation is that FAEE generation via CEL was responsible for pancreatic injury. Importantly, in vitro damage was reflected in pathological changes in a novel murine model of alcoholic AP, a significant development in an area thus far lacking a convenient and reliable means of inducing this disease. Although the association between alcohol and AP has long been recognised, our understanding of the underlying pathophysiology has been hampered by lack of an appropriate experimental model; no reliable model of AP has been made using application of ethanol alone^30^ and other agents have been required, including cholecystokinin^25^ ^31^ whose actions may be sensitised by alcohol.^25^ ^32^ In the current study, intraperitoneal injections of ethanol/POA to mice caused elevation of disease biochemical markers and development of extensive pancreatic necrosis, neutrophil infiltration and oedema. Such changes are consistent with acute damage observed in rats by intra-arterial administration of FAEEs^26^ or intravenous ethanol under conditions of OME inhibition.^3^ In accord with in vitro findings, 3-BCP prevented the increase of pancreatic POAEE in vivo and development of AP induced by ethanol/POA, indicating that alcohol-induced toxic effects are primarily mediated by FAEE production.

It has been suggested that OME may contribute to development of alcoholic AP via oxidative stress. For example, acute ethanol administration caused oxidative changes in rat pancreatic tissue,^33^ while high concentrations of acetaldehyde in vivo damaged rat and dog pancreas.^34^ In contrast to the toxic effects of POAEE, however, the OME metabolite acetaldehyde induced no increase in PAC death. This supports previous observations that acetaldehyde does not generate toxic Ca^2+^ elevations in PACs^12^ and actually increased mitochondrial NAD(P)H (data not shown) that would stimulate ATP production, consistent with lack of toxicity. Furthermore, generation of reactive oxygen species in exocrine pancreas may be protective, since this promotes activation of apoptosis rather than necrosis.^14^ ^16^ Important differences between NOME and OME have been documented that support this view. For example, whereas FAEEs activated NF-κB in PACs, acetaldehyde was inhibitory,^1^ while sustained elevation of acetaldehyde in ethanol-fed rats prevented hepatic inflammation and necrosis.^35^

FAEEs are reported to exert many detrimental effects on PACs, including premature activation of proteases and lipases capable of digesting subcellular structures, effects which may involve IP_3_ receptor-mediated Ca^2+^ release from acidic stores^36^ and increased fragility of lysosomes.^37^ Previously we have shown that toxic effects of FAEEs occur via Ca^2+^-dependent mitochondrial inhibition leading to a fall of ATP and necrosis.^13^ ^38^ ^39^ In the present study, blockade of OME transformed [Ca^2+^]_C_ signals induced by ethanol/POA from transient rises to toxic, sustained elevations and potentiated mitochondrial inhibition. Growing evidence indicates that mitochondrial dysfunction is a core pathological feature of AP^38^ ^40^ and an absence of a functional Na^+^/Ca^2+^ exchanger renders the PAC critically dependent upon ATP levels for Ca^2+^ homeostasis. Thus FAEE-induced ATP depletion would arrest Ca^2+^ extrusion via the plasmalemmal Ca^2+^-ATPase and refilling of internal stores via the sarco-endoplasmic reticulum Ca^2+^-ATPase, resulting in elevated [Ca^2+^]_C_; Ca^2+^ entry triggered by store depletion would further exacerbate mitochondrial Ca^2+^ overload. Here, use of a novel fluorescent probe indicated that FAEEs distribute to mitochondria, where breakdown occurs releasing fatty acids. This is in accord with biochemical measurements in cardiomyocytes, which demonstrated that >70% of FAEEs bound preferentially to mitochondria and underwent hydrolysis.^18^ Such FAEE hydrolytic activity has been demonstrated in the human pancreas,^41^ although the identity of the mitochondrial enzyme(s) responsible has yet to be clarified. Free fatty acids induce sustained [Ca^2+^]_C_ elevations and necrosis of PACs^12^ ^42^ and are known to uncouple mitochondria at concentrations as low as 5 µM.^18^ ^43^ ^44^ In the present study, hydrolysis of POA-Fluor was associated with sustained elevation of [Ca^2+^]_C_ and loss of NAD(P)H, consistent with mitochondrial inhibition as fatty acids are released.

Diverse enzymes can synthesise FAEEs, collectively known as FAEE synthases, including CEL, triglyceride lipase, lipoprotein lipase and acyl-coenzymeA:ethanol*O*-acyltransferase.^4^ These are poorly characterised, although the gene structure and cDNA sequence of human CEL has been elucidated^45^ with high enzyme expression reported in pancreas.^46^ ^47^ Immunofluorescence detected CEL in the granular, apical area of PACs under basal conditions. However, stimulation with cholecystokinin in vitro or caerulein in vivo redistributed the enzyme to the luminal area in pancreatic lobes, consistent with secretion. CEL enters the secretory pathway, and in adult humans, comprises approximately 4% of protein in pancreatic juice.^48^ However, pancreatic damage can release CEL into the blood^49^ with increased levels reported in patients with acute interstitial or necrotising pancreatitis, leading to the proposition that CEL may constitute a sensitive marker of AP severity.^11^ Furthermore, CEL redistributed outside of zymogen granules into the cytosolic compartment of necrotic PACs, with intense staining found around necrotic pancreatic lobules and in areas of fat necrosis in AP.^19^ In accord, in pancreatic lobules from mice treated with ethanol/POA in vivo, there was a diffuse intragranular and extragranular distribution of CEL throughout the PACs, apparent in damaged areas as early as 15 min after application of ethanol/POA. In an alcohol-rich and fatty acid–rich environment, localised elevation of CEL would enhance production of FAEEs capable of causing extensive tissue damage. In acutely intoxicated patients, FAEEs may attain levels as high as 115 µM/L,^2^ concentrations shown in the present study to elicit significant cellular necrosis.

Importantly, the damaging effects of ethanol and fat induced by promotion of NOME, both in vitro and in vivo, were significantly ameliorated by inhibition of CEL with 3-BCP, an agent previously shown to inhibit FAEE production and toxicity in cultured AR42J cells^6^, and now in pancreas. Although the inhibitory action of 3-BCP has been previously documented, use of molecular modelling has now demonstrated its likely mechanism of action. Structural analysis indicates stable binding of 3-BCP into the active site of CEL, via interaction with a Ser194 residue, that would compete with fatty acids and thus inhibit ethanol-mediated fatty acid esterification required for toxic FAEE generation. Identification of critical binding features suggests that rational design of specific modulators of CEL to block FAEE production is feasible, and candidate drugs may now be tested using a convenient and reliable model of alcoholic AP.

## Supplementary Material

Web supplement
