# Supplementary material for: Fatty acid ethyl ester synthase inhibition ameliorates ethanol-induced Ca2+-dependent mitochondrial dysfunction and acute pancreatitis
Source: Gut. 2013 Oct 25;63(8):1313–24. doi: 10.1136/gutjnl-2012-304058 (PMC4112447; doi:10.1136/gutjnl-2012-304058)
Supplement: Web supplement [file gutjnl-2012-304058-s1.pdf]

Supplementary Materials and Methods

Synthesis of POA-Fluor

To palmitoleic acid (254 mg, 1 mmol) was added oxalyl chloride (1.5 mL, excess). The mixture was stirred at room temperature for 2 h and excess oxylyl chloride was evaporated off. The crude product (palmitoleic acid chloride) was dissolved in ethyl acetate (5 mL) and fluorescein (400 mg, 1.20 mmol) was added. The mixture was stirred at room temperature for 4 h. Excess fluorescein was removed and the filtrate was purified by column chromatography (eluting with ethyl acetate) to afford the pure POA-Fluor in good yield (358mg, 63%). 1H-NMR (CDCl3): 8.01 (1H, m, Ar-H), 7.62 (2H, m, Ar-H), 7.06 (2H, m, Ar-H), 6.65 (6H, m, Ar-H), 5.35 (2H, m, HC=CH), 2.37 (2H, m, CH2), 2.02 (4H, m, 2xCH2), 1.67 (2H, m, CH2), 1.29 (16H, 8xCH2), 0.88 (3H, t, CH3). A schematic of the synthesis of POA-Fluor is shown in Supplementary Figure S1.

Acute Pancreatitis Induced by Fatty Acid Ethyl Ester

Male CD1 mice (30-35 g) were purchased from Charles River UK Ltd (Margate, UK) and housed at 23 ± 2°C under a 12-hr light/dark cycle with ad libitum access to standard laboratory chow and water. For 12 hrs before the start of the experiments, the animals were deprived of food but were allowed access to water ad libitum. Studies were conducted in compliance with the appropriate UK Home Office personal and project licence (40/3320), and with the Institutional ethical review processes of the University of Liverpool.

Initially to establish a novel alcoholic acute pancreatitis model induced by fatty acid ethyl ester (FAEE, FAEE-AP), the dose of ethanol was firstly optimised to 1.35

g/kg, which alone did not cause any obvious morphological changes in the pancreas. Palmitoleic acid ethyl ester (POAEE) was dissolved in pure ethanol to make stock solutions for injection. Mice received two intraperitoneal injection of POAEE (165 mg/kg) and ethanol (1.35 g/kg) at 1 hr interval based on the evidence that infusion of FAEE causes features of acute pancreatitis (AP) both in vivo and in vitro. To avoid local damage by ethanol to the peritoneal organs at the injection site, 200 μl of normal saline was injected immediately prior to the POAEE/ethanol injection. Analgesia was achieved by administration of 0.1 mg/kg buprenorphine hydrochloride (Temgesic, Reckitt and Coleman, Hull, England).

Induction of FAEE-AP by Fats and Ethanol

Subsequently a model was developed to more closely resemble the development of alcoholic acute pancreatitis, based on promotion of non-oxidative ethanol metabolism. Thus to demonstrate that endogenous FAEEs (i.e. synthesized from fat and ethanol by pancreatic FAEE synthase) could cause AP, oleic acid (OA, 165 mg/kg) or palmitoleic acid (POA) in combination with ethanol were also used. POA at doses of 10, 20, 80 and 150 mg/kg (equivalent molar concentration to 165 mg/kg POAEE or OA) and ethanol (1.35 kg/kg) were injected simultaneously. OA and ethanol (1.35 g/kg) were also injected simultaneously using the same regimen. Ethanol alone, and POA (150 mg/kg) alone dissolved in peanut oil were used as controls. Animals were sacrificed at various time points after the first injection for histopathology and severity marker analyses.

Induction of Caerulein (CER)-AP

In order to investigate the specificity of 3-benzyl-6-chloro-2-pyrone (3-BCP), another experimental model of acute pancreatitis was used; the caerulein (CER) hyperstimulation model [1]. Acute pancreatitis was induced in male (25-30 g) mice by 7 repeated intraperitoneal injections of CER (50 μg/kg/h) after which mice were sacrificed 12 h after first CER injection. 3-BCP (30 mg/kg) was administered at the third injection of CER whilst control animals received only saline.

Serum Amylase and IL-6 Levels

After sacrificing the mice, blood was allowed to clot naturally for 30 mins followed by centrifugation (1,500 g × 10 mins). The supernatants collected were tested at the Clinical Biochemistry Department in Royal Liverpool University Hospital, using a kinetic method [2] by Roche automated clinical chemistry analyzers (GMI, Leeds, UK). Serum IL-6 levels were tested by the ELISA method according to the instructions provided by the manufacturers (R&D, Abingdon, UK). Serum ethanol concentration was measured by an established enzymatic technique [3].

Pancreatic Water Content

The pancreata were weighed immediately following sacrifice of the mice and incubated at 90°C for 72 h. The fully dehydrated tissues were weighed again and the pancreatic water content was calculated as: wet weight-dry weight/wet weight × 100%.

Pancreatic Trypsin Activity

Pancreata were homogenised by a motorised homogeniser on ice in tissue homogenisation buffer pH 6.5, containing (in mM): MOPS 5, sucrose 250 and

magnesium sulphate 1. The resulting homogenates were centrifuged at 1500 g for 5 min, and 100 µl of each supernatant was added to a cuvette, containing peptide substrate Boc-Gln-Ala-Arg-MCA (Peptide, Osaka, Japan) dissolved in 1900 µl pH 8.0 assay buffer (in mM): Tris 50, NaCl 150, $CaCl_2$ 1 and 0.1 mg/ml bovine serum albumin. Trypsin activity was measured by fluorimetric assay using a Shimadzu RF-5000 spectrophotometer (Milton Keynes, UK). Samples were excited at 380 nm and emission collected at 440 nm [4, 5]. A standard curve was generated using purified human trypsin. Trypsin activity was expressed as fmol/mg protein or arbitrary units normalised to normal controls.

Pancreatic Myeloperoxidase Activity

Pancreatic myeloperoxidase (MPO) activity was tested by a modified method from Dawra *et al.* [6]. Myeloperoxidase activity was measured by using substrate 3,3′,5,5′-tetramethylbenzidine (TMB). Briefly, 20 µl of the supernatant was added into the assay mixture which consisted of 200 µL of phosphate buffer (100 mM, pH 5.4) with 0.5% HETAB, 20 µl TMB (20 mM in DMSO). This mixture was incubated at 37°C for 3 min, followed by the addition of 50 µL H_2O_2 (0.01%). This mixture was further incubated for 3 min. The difference of absorbance between 0 min and 3 min at 655 nm was calculated by a standard curve generated by human MPO using a plate reader. Pancreatic MPO activity was expressed as mU/mg protein or arbitrary units normalised to normal controls.

Western Blot Analysis

Protein extracted from mouse pancreatic acini was detected by Western Blot. Briefly the cells were washed with cold phosphate-buffered saline twice. The cells were

then lysated in RIPA buffer (50mM Tris-HCL pH8, 150mM NaCl, 0.5% Sodium deoxycholate, 1% Igepal CA-630 and 10% SDS). Different amounts of protein (40 µg, 20 µg, 10 µg) were sized-fractioned on 10% Mini-PROTEANR TGXTM Precast Gel (Bio-Rad Laboratories) and transferred to Trans-BlotR TurboTM Mini PVDF Transfer Packs (Bio-Rad Laboratories) using a Trans-BlotR TurboTM Transfer Starter System (Bio-Rad Laboratories). The membrane was incubated overnight at 4oC with anti-CEL (ab79131, 1:500, Abcam), and then probed with horseradish peroxidase-conjugated secondary antibodies. Anti- β-actin (1:5000, Sigma) was used as loading controls. Immunoblots were detected by enhanced chemiluminescence.

POAEE Extraction/Isolation From Plasma/Whole Pancreas

Immediately following the sacrifice of each mouse, blood was collected into an EDTA tube and the pancreas removed and placed in 1ml of hexane (placed on ice). Blood was centrifuged at 1500 x g for 10mins and the plasma layer collected. 200 µL of plasma was transferred to a fresh tube and 1ml of ice-cold acetone added, vortexed for 1min and placed on ice for 15mins. The plasma samples were then centrifuged at 600 x g and the supernatant transferred in to a fresh 15ml glass tube. The whole pancreas was homogenised in 1ml of hexane and transferred to a fresh 15ml glass tube. 2ml of ice-cold acetone was then added to the homogenate and vortexed for 1min and centrifuged at 600 x g for 10mins. The supernatant was removed and placed into a fresh 15ml glass tube. Ethyl dodecanoate (E12:0) was used as internal standard and 10 µL (1 nmole) was added to each sample. The FAEEs were then extracted by adding 2 x 4mL volumes of hexane (vortexing and centrifuging at 600 x g in between volumes) and the solvent layer transferred into a

fresh glass tube and then dried under nitrogen to 300 µL. FAEEs were then isolated using solid-phase extraction with an aminopropyl column (1mL/100mg, Chromabond, FisherScientific, UK) as described by Bernhardt et al [7]. Following which the samples were again dried under nitrogen to a final volume of 100 µL.

Quantification of POAEE

POAEE in plasma and pancreas was determined by gas chromatography–mass spectrometry (GC-MS) using a Trace Gas-Chromatograph coupled to a PolarisQ Ion-Trap mass spectrometer (ThermoScientific, Hemel Hempstead, UK) equipped with a SP-2330 capillary column (Supelcowax, Sigma, UK). The oven temperature was maintained at 160 °C for 1 min, increased to 180 °C at 3 °C/min, then further increased to 210 °C at 6 °C/min and held for 5 min. The injector and mass spectrometer were maintained at 250 and 215 °C, respectively, with a carrier gas flow rate of 1 L/min throughout. Single ion monitoring was performed, with ions m/z 55 and 236 monitored for ethyl palmitoleate (E16:1) and m/z 88 and 157 for ethyl dodecanoate (E12:0). POAEE levels in both plasma and pancreas were quantified by interpolation of the slope generated from a peak area calibration curve with increasing concentrations of E16:1 (0.5 to 10 pmole/µL) compared to a fixed concentration of the internal standard (E12:0).

Carboxylester Lipase (CEL) Inhibition Assay

CEL activity was evaluated by measuring the hydrolysis of *p*-nitrophenylacetate (PNPA), a substrate of this enzyme, as described previously [8] and the inhibitory effect of 3-BCP (1-10 µM) investigated. Isolated murine pancreatic acinar cells from CD1 mice were prepared as described earlier (Main Methods Section) and cell

pellets re-suspended in Dulbecco's modified Eagle medium containing 10 mM glucose, 2 mM L-glutamine and 2 mM sodium pyruvate. A cell count was determined using the NucleoCounter system (Chemometec) enabling seeding at 0.5 x 10^6 viable cells per 1.5 ml micro-centrifuge tube. The cells were incubated at 37^oC for 5 minutes with or without 3-BCP (1, 5 and 10 µM); conditions were repeated in duplicate. The cells were harvested by centrifugation at 600g for 5 minutes, washed with 0.05 M phosphate buffer (pH 7.4) and homogenised in the same buffer. The PNPA-hydrolysing activities were measured as previously described [8], using total *p*-nitrophenol formation linked to hydrolysis of cholesteryl oleate in the presence of sodium deoxycholate, and the rate of activity expressed as nmol/min/mg protein. Protein was measured using a BCA (bicinchoninic acid) assay (Pierce) with bovine serum albumin as standard.

ATP Measurement

Confocal microscopy experiments were performed on murine pancreatic acinar cells to determine changes of intracellular ATP concentrations after loading with Magnesium Green (Mg Green-AM; 4μM) for 30 min at room temperature, as previously described [9]. Decrease of intracellular ATP is seen as an increase in Mg Green fluorescence (excitation 476 nm, emission 500–550 nm) in the presence of a calcium- and magnesium-free extracellular solution.

Supplementary Figure Legends

Supplementary Figure 1. A schematic showing the synthesis and chemical structure of the palmitoleic acid-fluorescein conjugate (POA-Fluor), a novel probe for fatty acid ethyl ester activity. The fluorescein molecule is attached at the carboxylic acid moiety of POA, analogous to the ethyl ester linkage present in the non-oxidative ethanol metabolite palmitoleic acid ethyl ester (POAEE), and is released intracellularly in isolated pancreatic acinar cells via the action of hydrolase enzymes (Figure 3).

Supplementary Figure 2. Localisation of carboxylester lipase (CEL) in isolated murine pancreatic lobules. (A) Light-transmitted and fluorescent confocal images of intact pancreatic tissue obtained from mice treated with two hourly intraperitoneal injections of cerulein (0.2 µg/kg/h), which has previously been shown to stimulate secretion [10], and sacrificed 30 minutes after the second injection. CEL (*green*) is located predominantly in the lumen between the acinar clusters, indicative of secretion. Nuclei were co-stained with Hoechst 33342 (*blue*). This is in contrast to the generalised CEL distribution observed in intact pancreas obtained from mice treated with an POA/ethanol combination to induce in vivo alcoholic AP (Figure 5C). (B) Autoradiograph overlaid on a Western blot membrane showing specificity of carboxylester lipase antibody. Protein was extracted from purified murine pancreatic acini and separated by SDS-PAGE. The CEL antibody used was specific for a single band on the Western blot indicating a protein of approximately 70KDa. This is consistent with the known migration of the CEL protein and therefore indicates a high

level of specificity for the antibody. (C) Light-transmitted and fluorescent confocal images of intact pancreatic tissue obtained from control mice (*upper panels*) and mice treated with ethanol/POA combination (*lower panels*) to induce alcoholic AP (i.p. ethanol (1.35 g/kg) and POA (150 mg/kg) sacrificed at an early time-point, 15 mins after the final ethanol/POA injection. An apical CEL (*green*) distribution was observed in control animals, whereas a diffuse distribution of CEL was observed in areas of damage from intact pancreatic lobules obtained from mice treated with ethanol/POA combination. Nuclei were co-stained with Hoechst 33342 (*blue*). (Data representative of 10 samples from 4 mice (treated) and 6 samples from 4 mice (control)).

Supplementary Figure 3. Time course of FAEE-AP induced by ethanol with POA. FAEE-AP was induced by two intraperitoneal injections of ethanol (1.35 g/kg) and POA (80 mg/kg). For serum ethanol (EtOH) concentration measurements, mice were sacrificed at 1.5, 2, 3, 6 and 12 hr after the first injection, otherwise mice were sacrificed at 24 hr. Time course of (A) serum ethanol concentration, (B) Serum amylase, (C) Pancreatic water content, (D) Pancreatic trypsin activity, and (F) Pancreatic MPO activity (normalised). Values are mean ± SE of 4-6 mice.

Supplementary Figure 4. Co-administration of ethanol and palmitoleic acid ethyl ester (POAEE) or oleic acid (OA) causes AP. Fatty acid ethyl ester-induced acute pancreatitis (FAEE-AP) was induced by two intraperitoneal injections of ethanol (1.35 g/kg) with POAEE (165 mg/kg) or OA (165 mg/kg), mice were sacrificed at 2, 4, 8, 12, 24 and 48 hr after first injection. (A) Representative H&E images of histology slides from pancreas of mice treated with ethanol and POAEE. (B)

Representative H&E images of histology slides from pancreas of mice treated with OA/ethanol. Magnification, × 200.

Supplementary Figure 5. Pancreatic levels of palmitoleic acid ethyl ester (POAEE) are increased in fatty acid ethyl ester-induced acute pancreatitis (FAEE-AP) and inhibited by 3-benzyl-6-chloro-2-pyrone (3-BCP). FAEE-AP was induced by two intraperitoneal injections of ethanol (EtOH; 1.35 g/kg) with POA (50 mg/kg) and mice were sacrificed at 30 mins, 1 and 2 hrs after the first injection for POAEE measurement. (A) Graph showing increased POAEE levels in the pancreas at 30 minutes after the last ethanol/POA injection, which declined at 1 and 2 hrs but remained significantly greater than control levels. Administration of 3-BCP (30 mg/kg) significantly inhibited the increase in POAEE at 30 mins. However, plasma levels of POAEE were not significantly elevated in the FAEE-AP model consistent with a generation of FAEEs within the pancreas by carboxylester lipase (CEL). (Data are mean ± SE of 6 mice per group. *$p < 0.05$ compared to both saline and POA controls, $^{\#}p < 0.05$ compared to the POA/Ethanol group). (C) Inhibition of CEL activity by 3-BCP. The rate of CEL-induced hydrolysis of *p*-nitrophenylacetate (PNPA) in isolated murine pancreatic acinar cells was concentration-dependently inhibited by 3-BCP (1 - 10 µM). Data are expressed as the mean ± SE of 10 observations, 5 mice; *p < 0.05 compared to control).

Supplementary Figure 6. Lack of inhibitory action of 3-benzyl-6-chloro-2-pyrone (3-BCP) in a caerulein (CER) hyperstimulation model of AP. CER (50 µg/kg/h) was given as 7 intraperitoneal injections and 3-BCP (30 mg/kg) administered at the third injection of CER. Mice were sacrificed 12 h after first CER injection. Parameters of

acute pancreatitis (A) Histology score, (B) Oedema, (C) Inflammation, (D) Necrosis, (E) Serum amylase, (F) Pancreatic trypsin and (G) Pancreatic myeloperoxidase (MPO) were not significantly different between 3-BCP-treated (*cyan*) and control (*black*) groups (Data are mean ± SE of 4 mice per group).

Supplementary Figure 7. Effects of 3-benzyl-6-chloro-2-pyrone (3-BCP) and extracellular Ca^{2+} removal on $[Ca^{2+}]_C$ signals in murine pancreatic acinar cells. Typical traces showing the effects of 3-BCP (10 μmol/L) on (A) basal $[Ca^{2+}]_C$ (n = 39), (B) oscillatory rises of $[Ca^{2+}]_C$ induced by cholecystokinin (CCK; 10 pmol/L) (n = 12), (C) sustained rise of $[Ca^{2+}]_C$ induced by cholecystokinin (10 nmol/L) (n = 32), (D) sustained rise of $[Ca^{2+}]_C$ induced by palmitoleic acid (POA; 100 μmol/L) (n = 7), (E) oscillatory rises of $[Ca^{2+}]_C$ induced by POA/ethanol combination (POA; 20 μmol/L and EtOH 10 mmol/L) (n = 7), and the effects of extracellular Ca^{2+} removal on (F) oscillatory (*grey;* n = 8) and sustained (*black;* n = 3) rises of $[Ca^{2+}]_C$ induced by POA/ethanol combination (POA; 20 μmol/L and EtOH 10 mmol/L), and (G) sustained rise of $[Ca^{2+}]_C$ induced by POA/ethanol combination with 4-methylpyrazole (4-MP; 100 μmol/L) (n = 11).

Supplementary Figure 8. Protective effects of 3-benzyl-6-chloro-2-pyrone (3-BCP) on POA/ethanol-induced changes of mitochondrial membrane potential ($\Delta\psi_M$), reduced nicotinamide adenine dinucleotide (phosphate) (NAD(P)H) and adenosine triphosphate (ATP) in murine pancreatic acinar cells. The effects of 3-BCP (10 μmol/L, *cyan*) on POA/ethanol-induced (*wine*) decreases of (A) $\Delta\psi_M$ and (C) NAD(P)H (n = 11) and 3-BCP alone in control experiments (B and D, respectively) (n = 9). (Values are mean ± SE). Typical traces showing the inhibitory effects of 3-BCP

(10 μmol/L) on POA/ethanol-induced increases of Magnesium Green fluorescence, reflecting a decrease of intracellular ATP concentration (E; n = 23), with no effect in control experiments in the absence of POA/ethanol (F; n = 23). Addition of the protonophore carbonyl cyanide 3-chlorophenylhydrazone (CCCP; 10 μmol/L) was used to cause a maximal effect as previously described [9].

REFERENCES

1 Saluja AK, Lerch MM, Phillips PA, *et al.* Why Does Pancreatic Overstimulation Cause Pancreatitis? *AnnuRevPhysiol* 2006.

2 Lorentz K. Approved recommendation on IFCC methods for the measurement of catalytic concentration of enzymes. Part 9. IFCC method for alpha-amylase (1,4-alpha-D-glucan 4-glucanohydrolase, EC 3.2.1.1). International Federation of Clinical Chemistry and Laboratory Medicine (IFCC). Committee on Enzymes. *Clin Chem Lab Med* 1998;**36**:185-203.

3 Bucher T, Redetzki H. [Specific photometric determination of ethyl alcohol based on an enzymatic reaction]. *Klin Wochenschr* 1951;**29**:615-6.

4 Nathan JD, Romac J, Peng RY, *et al.* Transgenic expression of pancreatic secretory trypsin inhibitor-I ameliorates secretagogue-induced pancreatitis in mice. *Gastroenterology* 2005;**128**:717-27.

5 Kawabata S, Miura T, Morita T, *et al.* Highly sensitive peptide-4-methylcoumaryl-7-amide substrates for blood-clotting proteases and trypsin. *European journal of biochemistry / FEBS* 1988;**172**:17-25.

6 Dawra R, Ku YS, Sharif R, *et al.* An improved method for extracting myeloperoxidase and determining its activity in the pancreas and lungs during pancreatitis. *Pancreas* 2008;**37**:62-8.

7 Bernhardt TG, Cannistraro PA, Bird DA, *et al.* Purification of fatty acid ethyl esters by solid-phase extraction and high-performance liquid chromatography. *J Chromatogr B Biomed Appl* 1996;**675**:189-96.

8 Kaphalia BS, Mericle KA, Ansari GA. Mechanism of differential inhibition of hepatic and pancreatic fatty acid ethyl ester synthase by inhibitors of serine-esterases: in vitro and cell culture studies. *ToxicolApplPharmacol* 2004;**200**:7-15.

9 Criddle DN, Murphy J, Fistetto G, *et al.* Fatty Acid Ethyl Esters Cause Pancreatic Calcium Toxicity via Inositol Trisphosphate Receptors and Loss of ATP Synthesis. *Gastroenterology* 2006;**130**:781-93.

10 Saluja AK, Saluja M, Printz H, *et al.* Experimental pancreatitis is mediated by low-affinity cholecystokinin receptors that inhibit digestive enzyme secretion. *Proc Natl Acad Sci U S A* 1989;**86**:8968-71.

Figure S1

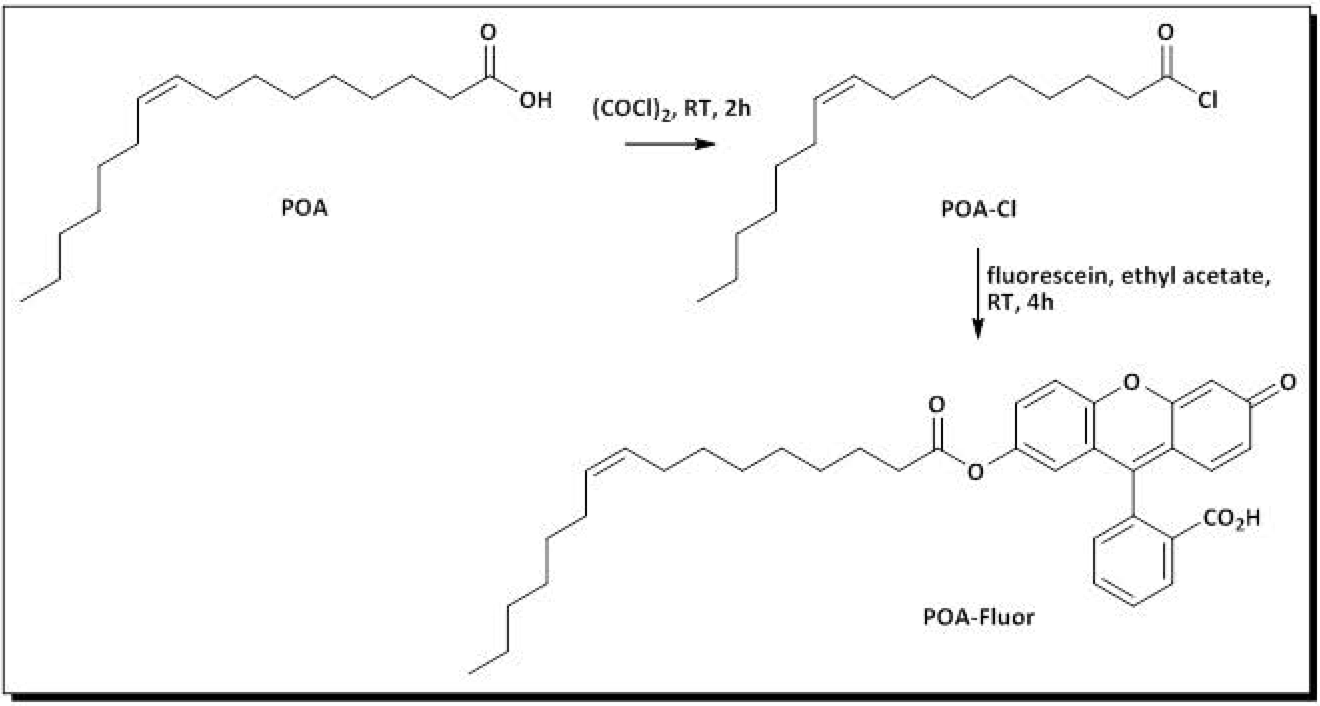
O
OH
POA
(COCl)2, RT, 2h
O
Cl
POA-Cl
fluorescein, ethyl acetate,
RT, 4h
O
O
O
O
CO2H
POA-Fluor

Figure S2

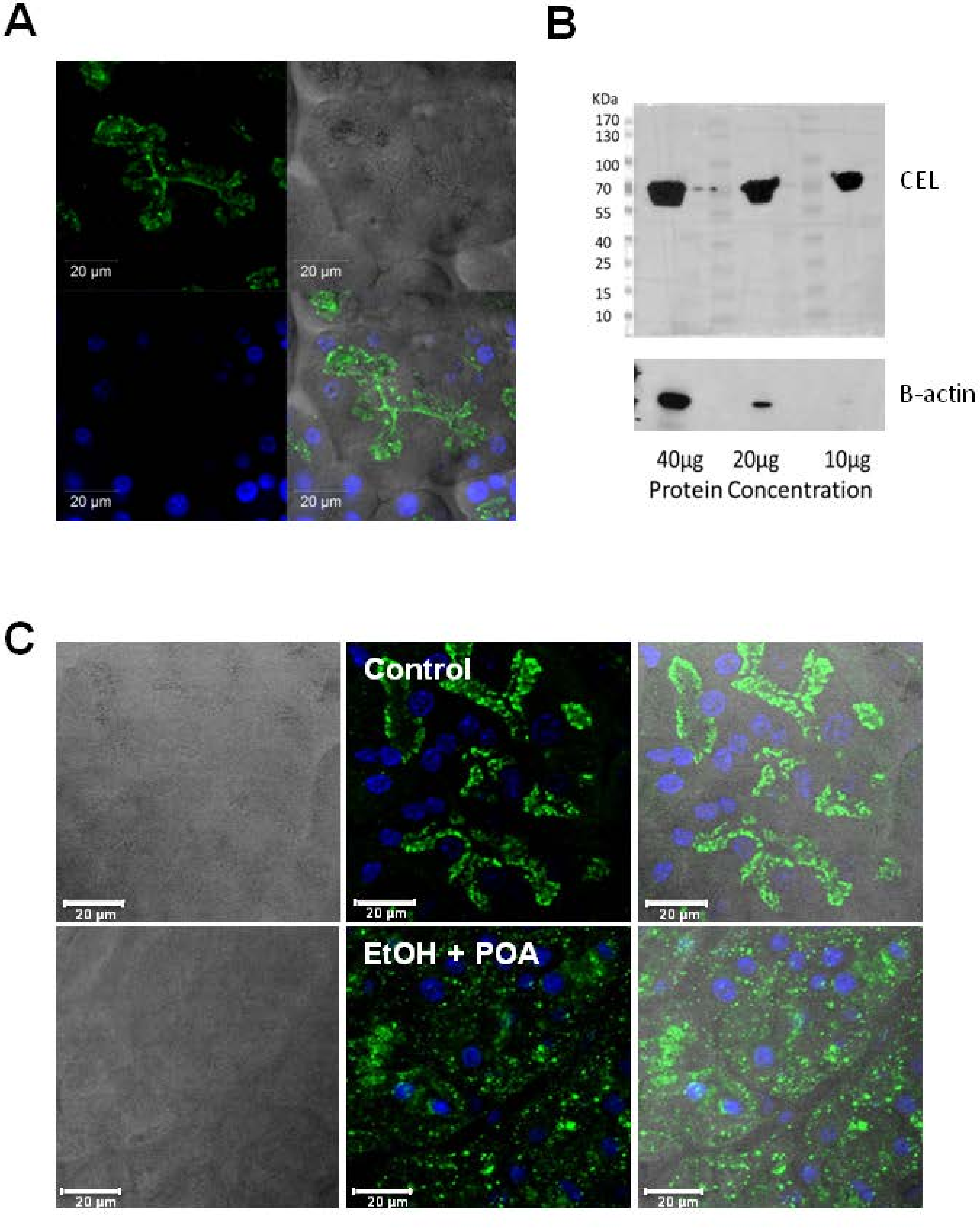
A
B
C
20 µm
KDa
170
130
100
70
55
40
25
15
10
CEL
B-actin
40µg
20µg
10µg
Protein Concentration
Control
EtOH + POA

Figure S3

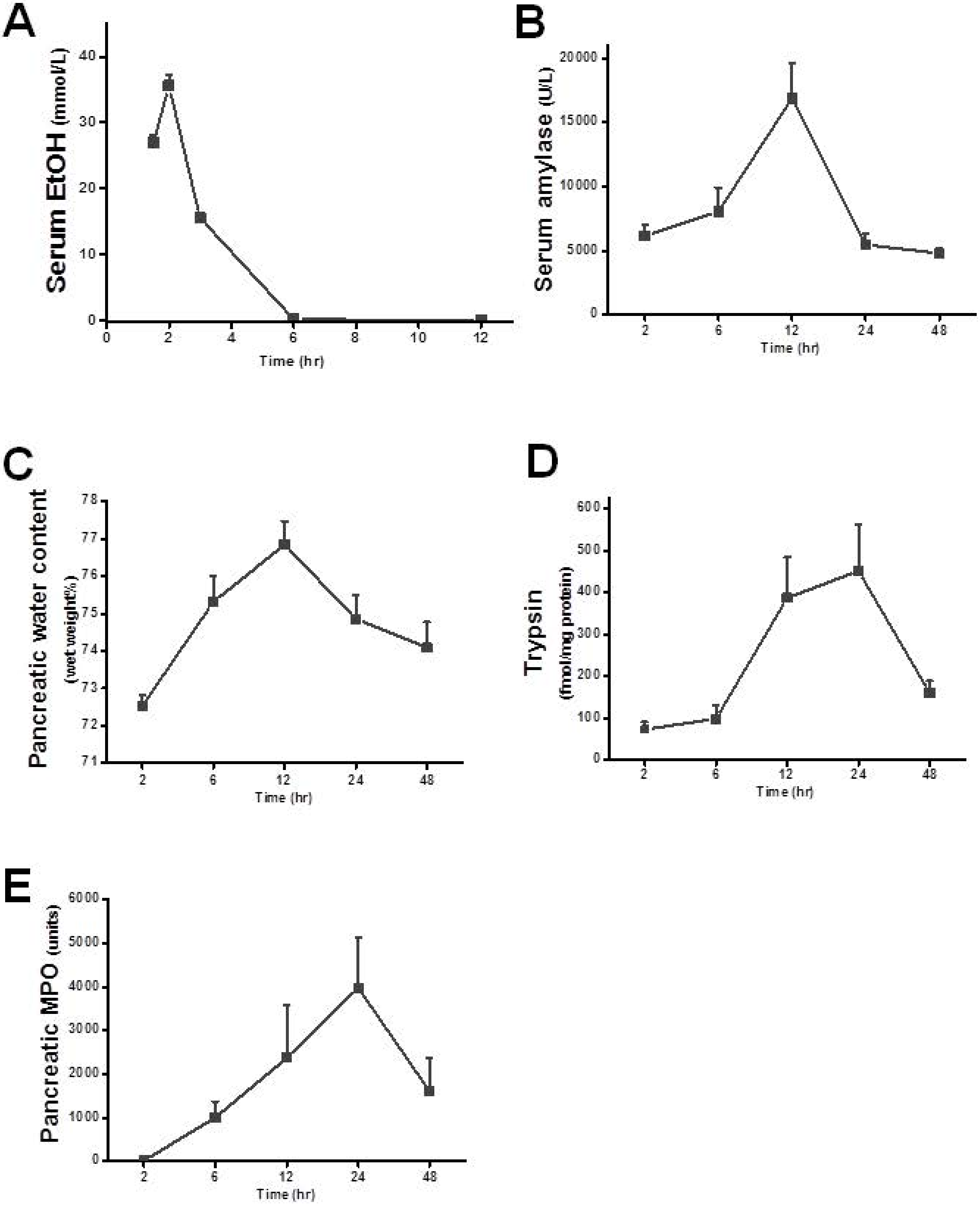
A
Serum EtOH (mmol/L)
40
30
20
10
0
0
2
4
6
8
10
12
Time (hr)
B
Serum amylase (U/L)
20000
15000
10000
5000
0
2
6
12
24
48
Time (hr)
C
Pancreatic water content
(wet weight%)
78
77
76
75
74
73
72
71
2
6
12
24
48
Time (hr)
D
Trypsin
(fmol/mg protein)
600
500
400
300
200
100
0
2
6
12
24
48
Time (hr)
E
Pancreatic MPO (units)
6000
5000
4000
3000
2000
1000
0
2
6
12
24
48
Time (hr)

Figure S4

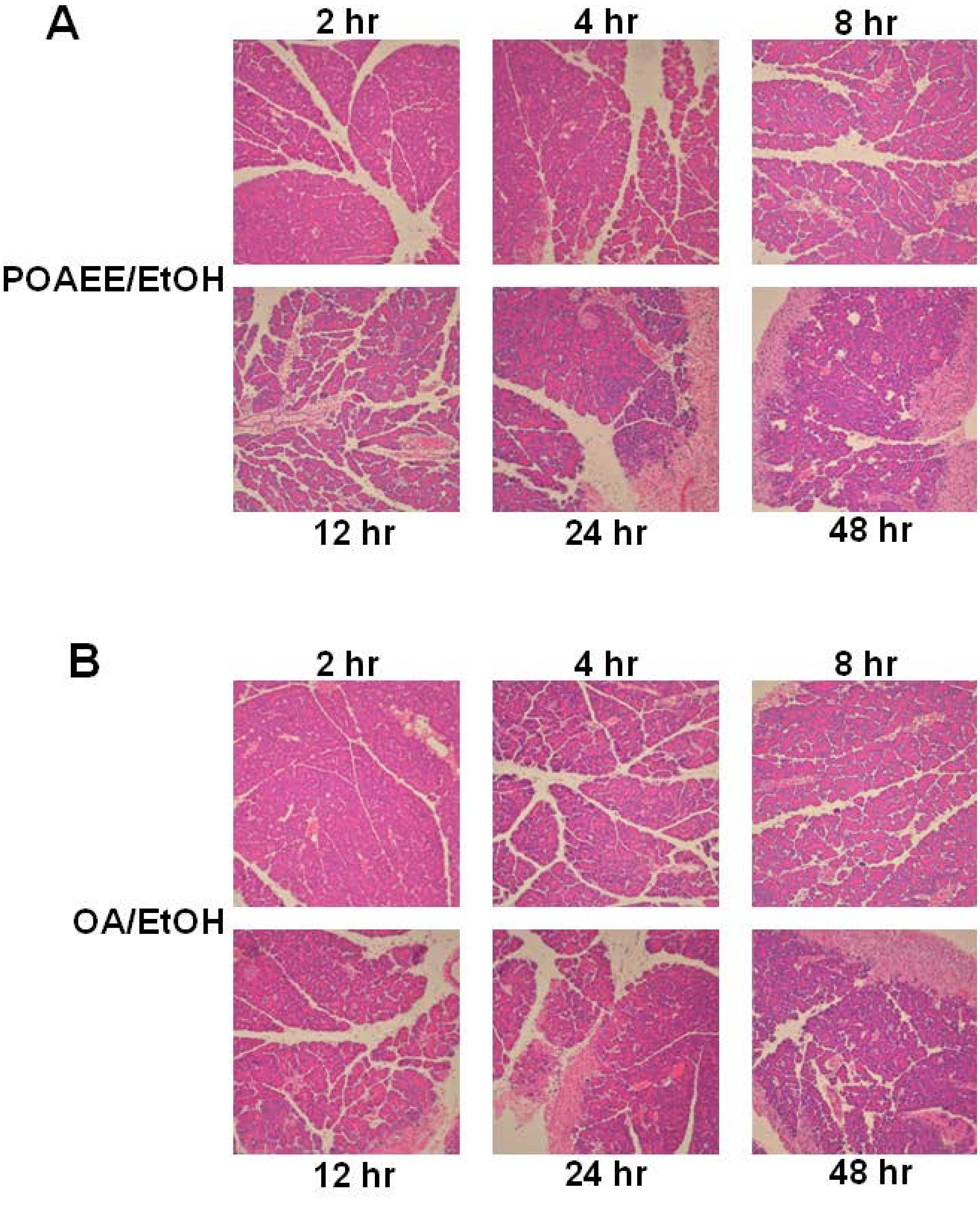
A
2 hr
4 hr
8 hr
POAEE/EtOH
12 hr
24 hr
48 hr
B
2 hr
4 hr
8 hr
OA/EtOH
12 hr
24 hr
48 hr

Figure S5

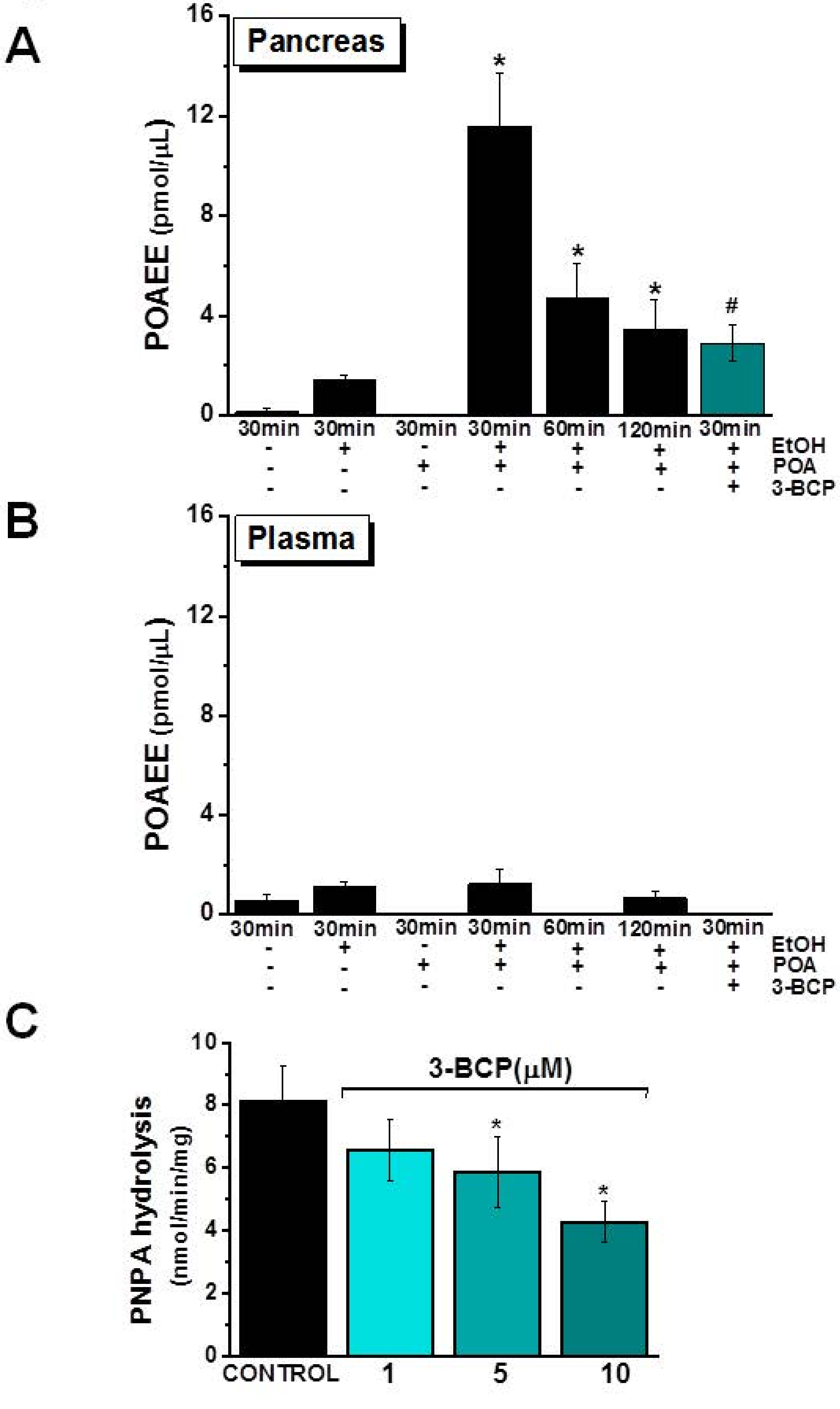
A
Pancreas
POAEE (pmol/μL)
16
12
8
4
0
*
*
*
#
30min 30min 30min 30min 60min 120min 30min
- + - + + + + EtOH
- - + + + + + POA
- - - - - - + 3-BCP
B
Plasma
POAEE (pmol/μL)
16
12
8
4
0
30min 30min 30min 30min 60min 120min 30min
- + - + + + + EtOH
- - + + + + + POA
- - - - - - + 3-BCP
C
PNPA hydrolysis (nmol/min/mg)
10
8
6
4
2
0
3-BCP(μM)
*
*
CONTROL 1 5 10

Figure S6

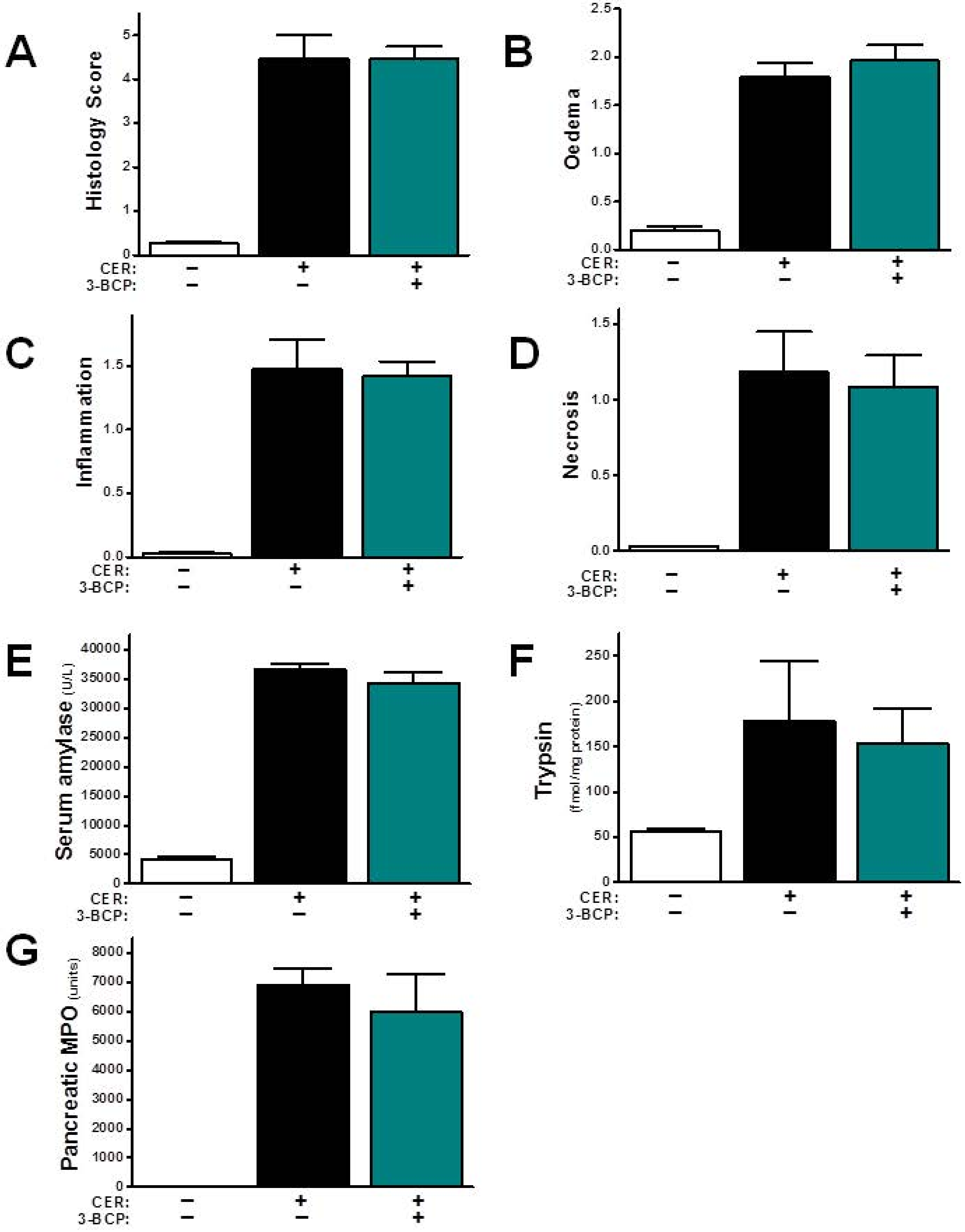
A
Histology Score
B
Oedema
C
Inflammation
D
Necrosis
E
Serum amylase (U/L)
F
Trypsin (fmol/mg protein)
G
Pancreatic MPO (units)
CER:
3-BCP:

Figure S7

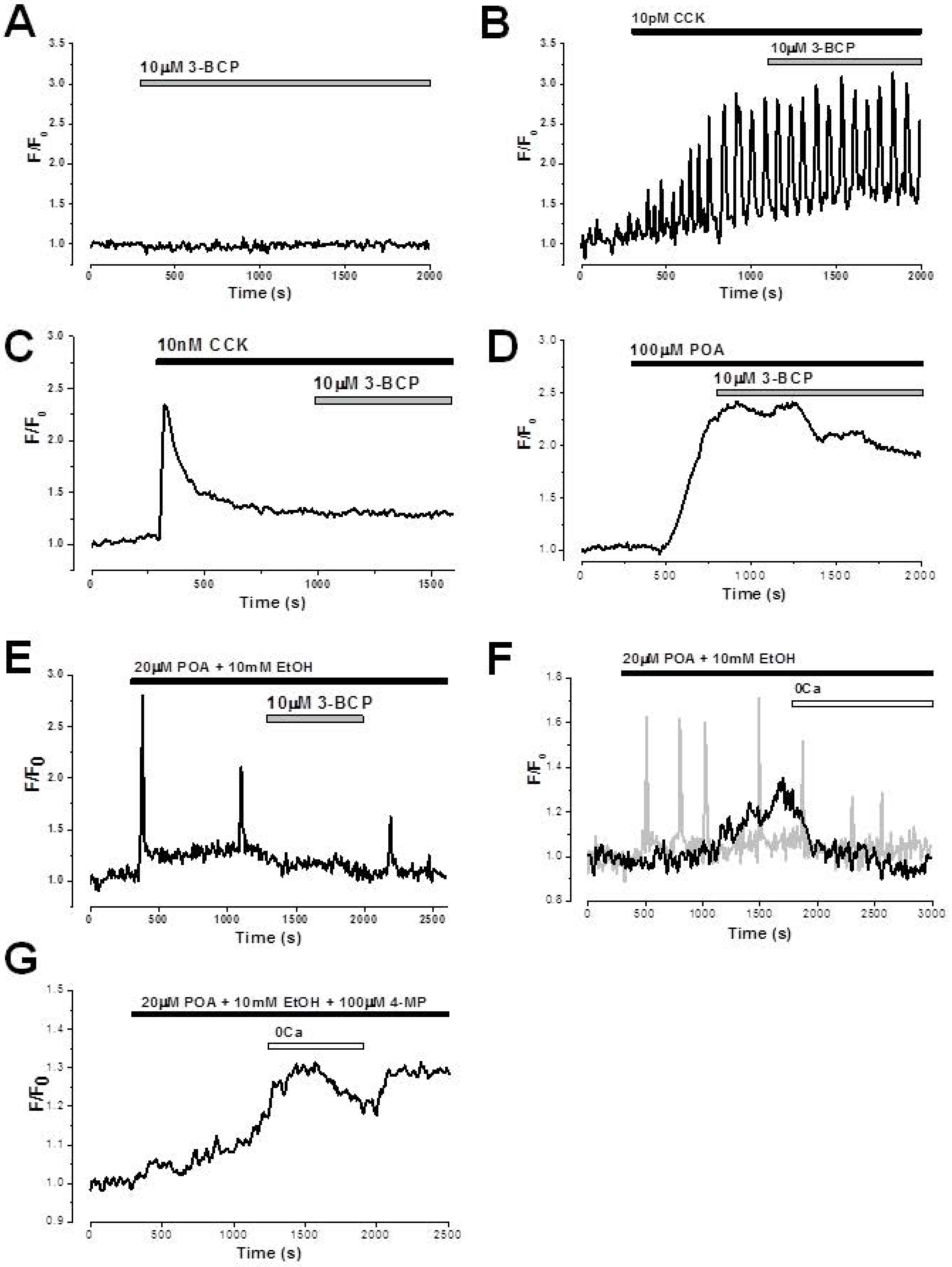
A
10µM 3-BCP
F/F0
Time (s)
B
10pM CCK
10µM 3-BCP
F/F0
Time (s)
C
10nM CCK
10µM 3-BCP
F/F0
Time (s)
D
100µM POA
10µM 3-BCP
F/F0
Time (s)
E
20µM POA + 10mM EtOH
10µM 3-BCP
F/F0
Time (s)
F
20µM POA + 10mM EtOH
0Ca
F/F0
Time (s)
G
20µM POA + 10mM EtOH + 100µM 4-MP
0Ca
F/F0
Time (s)

Figure S8

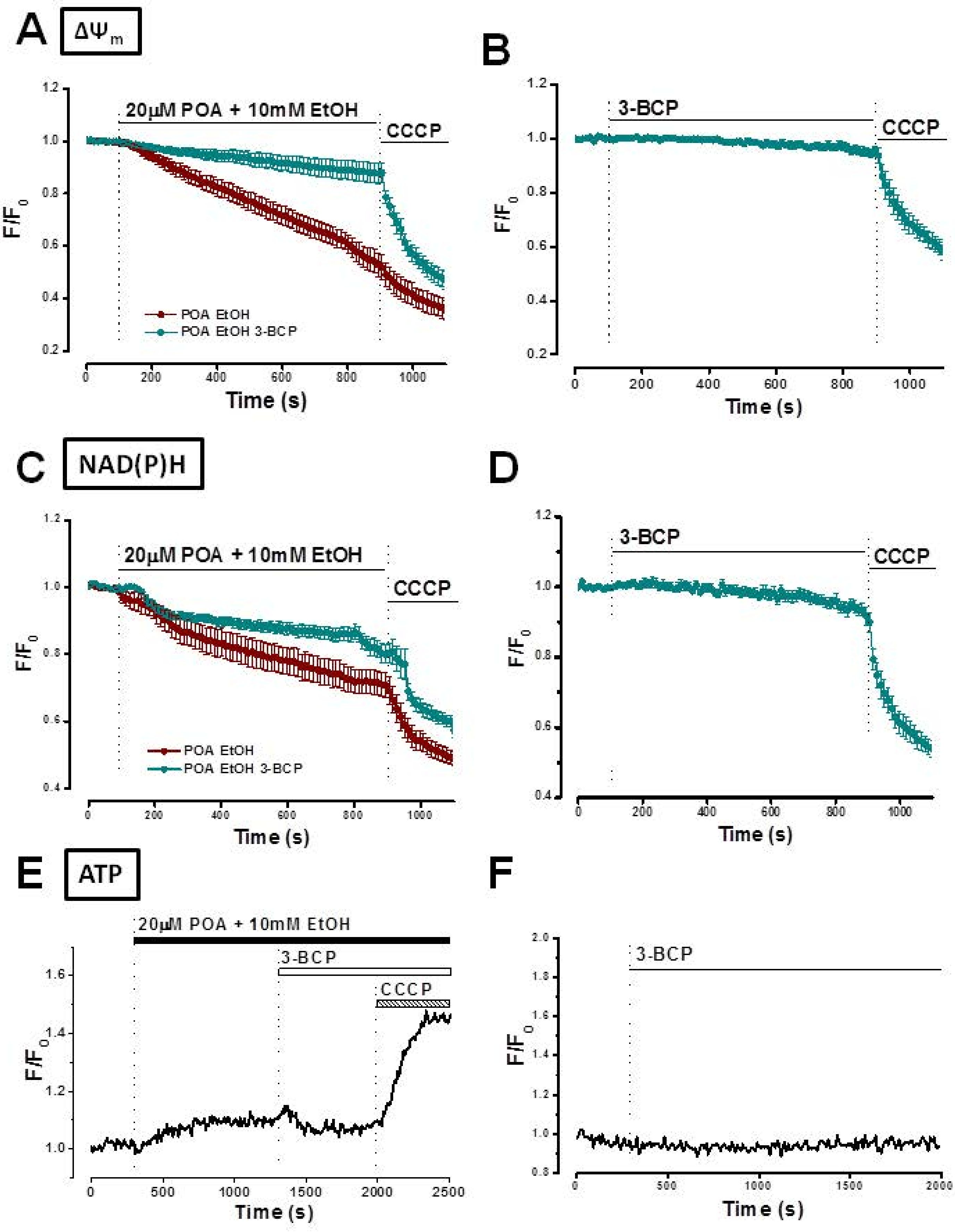

A
ΔΨm
20μM POA + 10mM EtOH
CCCP
POA EtOH
POA EtOH 3-BCP
F/F0
Time (s)
B
3-BCP
CCCP
F/F0
Time (s)
C
NAD(P)H
20μM POA + 10mM EtOH
CCCP
POA EtOH
POA EtOH 3-BCP
F/F0
Time (s)
D
3-BCP
CCCP
F/F0
Time (s)
E
ATP
20μM POA + 10mM EtOH
3-BCP
CCCP
F/F0
Time (s)
F
3-BCP
F/F0
Time (s)
